# Histone H4 dosage modulates DNA damage response in the pathogenic yeast *Candida glabrata* via homologous recombination pathway

**DOI:** 10.1371/journal.pgen.1008620

**Published:** 2020-03-05

**Authors:** Kundan Kumar, Romila Moirangthem, Rupinder Kaur

**Affiliations:** 1 Laboratory of Fungal Pathogenesis, Centre for DNA Fingerprinting and Diagnostics, Hyderabad, Telangana, India; 2 Graduate Studies, Manipal Academy of Higher Education, Manipal, Karnataka, India; Oregon State University, UNITED STATES

## Abstract

*Candida glabrata*, a nosocomial fungal bloodstream pathogen, causes significant morbidity and mortality in hospitals worldwide. The ability to replicate in macrophages and survive a high level of oxidative stress contributes to its virulence in the mammalian host. However, the role of DNA repair and recombination mechanisms in its pathobiology is still being discovered. Here, we have characterized the response of *C*. *glabrata* to the methyl methanesulfonate (MMS)-induced DNA damage. We found that the MMS exposure triggered a significant downregulation of histone H4 transcript and protein levels, and that, the damaged DNA was repaired by the homologous recombination (HR) pathway. Consistently, the reduced H4 gene dosage was associated with increased HR frequency and elevated resistance to MMS. The genetic analysis found CgRad52, a DNA strand exchange-promoter protein of the HR system, to be essential for this MMS resistance. Further, the tandem-affinity purification and mass spectrometry analysis revealed a substantially smaller interactome of H4 in MMS-treated cells. Among 23 identified proteins, we found the WD40-repeat protein CgCmr1 to interact genetically and physically with H4, and regulate H4 levels, HR pathway and MMS stress survival. Controlling H4 levels tightly is therefore a regulatory mechanism to survive MMS stress in *C*. *glabrata*.

## Author summary

The cellular hereditary material DNA is present in a compact ordered form in eukaryotic cells which involves its winding around an octamer of four basic histone proteins, H2A, H2B, H3 and H4. DNA-protein (including histones) complexes form chromatin, with the chromatin structure, open or closed, modulating gene expression. Any change in histone levels impacts chromatin architecture and functions. Here, we have studied the effect of diminished histone H4 levels on viability, DNA damage response and virulence of the pathogenic yeast *Candida glabrata*. *C*. *glabrata*, a constituent of the normal microflora of healthy humans, causes both superficial and invasive infections in immunocompromised individuals. Despite it being the second most common cause of *Candida* bloodstream infections in USA after *C*. *albicans*, its pathogenesis determinants are yet to deciphered in full. We report that the reduced histone H4 gene dosage in *C*. *glabrata* results in elevated resistance to the DNA alkylating agent, methyl methanesulfonate, increased homologous recombination (HR) and attenuated virulence. We also show that the H4 interacting protein CgCmr1 regulates HR probably through maintaining H4 levels. Overall, our data underscore the H4 protein abundance as a cue to express virulence factors and regulate DNA metabolism in pathogenic fungi.

## Introduction

Chromatin architecture is pivotal to the nucleic acid-regulated processes including replication, recombination, DNA repair and transcription [1,2]. Chromatin consists of nucleosomal arrays, with each nucleosome containing 146 bp DNA wrapped around a histone octamer [3]. The histone octamer is comprised of two molecules of each of four core histones, H2A, H2B, H3, and H4, with H3-H4 tetramer surrounded on either side by a H2A-H2B heterodimer [3]. The stoichiometry of histone proteins is important for proper chromatin structure and functions [4,5]. Histones are encoded by multiple genes, and histone synthesis is coupled with replication in *Saccharomyces cerevisiae* [6,7]. The tandem-repeat-structural organization of histone genes with shared regulatory sequences is thought to contribute to their coordinated expression [7,8].

*Candida glabrata* is an opportunistic human fungal pathogen of high clinical importance which accounts for up to 30% of *Candida* bloodstream infections [9–11]. The crude mortality rate associated with invasive *C*. *glabrata* infections varies from 33–46% [12–14]. *C*. *glabrata* also causes mucosal infections including oral and vaginal thrush [15–17]. *C*. *glabrata* is a haploid budding yeast, and its known virulence traits include two multigene families coding for cell wall adhesins and cell surface-associated aspartyl proteases, adherence to host tissues, biofilm formation on biotic and abiotic surfaces, intracellular replication and elevated resistance to oxidative, thermal and acid stress [18].

Evolutionarily, *C*. *glabrata* is closer to *S*. *cerevisiae* than to the most prevalent *Candida* species, *C*. *albicans* [19]. The *S*. *cerevisiae* genome harbours two copies of histone H3 (Hht)- and H4 (Hhf)-encoding genes, that are organized as two unlinked *HHT1-HHF1* and *HHT2-HHF2* gene pairs [6]. Notably, both loci encode identical histone H3 and H4 proteins [20], and one H3-H4 gene locus is required for cell viability [21]. Owing to the phylogenetic relatedness between *C*. *glabrata* and *S*. *cerevisiae*, chromatin organization, DNA damage repair and stress response mechanisms are presumed to be similar between these two yeasts [22–24].

*C*. *glabrata* is capable of survival and replication in murine and human macrophages [24–26]. The intracellular proliferation of *C*. *glabrata* is dependent upon its ability to remodel its chromatin, reprogram its carbon metabolism and induce autophagy [24]. Macrophage-internalized *C*. *glabrata* cells have been reported to display non-tandem expression of core histones. While levels of H1, H2A, H2B and H3 proteins were increased upon macrophage internalization, H4 protein levels were reduced [24]. This discordant expression of core histones is likely to perturb histone pair stoichiometry in macrophage-ingested *C*. *glabrata* cells.

Reduced dosage of histone H4 in *C*. *albicans* has previously been associated with growth and filamentation defects [27]. Similarly, histone H4 depletion led to defective chromosome segregation and cell cycle arrest in *S*. *cerevisiae* [28]. Since the ability of *C*. *glabrata* cells to proliferate intracellularly is pivotal to its pathogenesis, we here have investigated the role of histone H4 in the DNA damage response and virulence of *C*. *glabrata*. We show for the first time that the reduced H4 dosage is linked with increased homologous recombination and better survival of MMS (methyl methanesulfonate) stress. Additionally, we report that the MMS stress response in *C*. *glabrata* consists of diminished H4 transcript and protein levels, fewer number of protein interactors of the histone H4 and differential transcriptional regulation of transport, aerobic respiration and ergosterol biosynthetic genes. Lastly, we identified the WD40-repeat protein CgCmr1 as the H4 interactor, and demonstrated it to be an important component of the MMS-induced DNA damage response system.

## Results

### *C*. *glabrata* strains lacking histone H4 genes display differential susceptibility to methyl methanesulfonate

**Fig 1:**
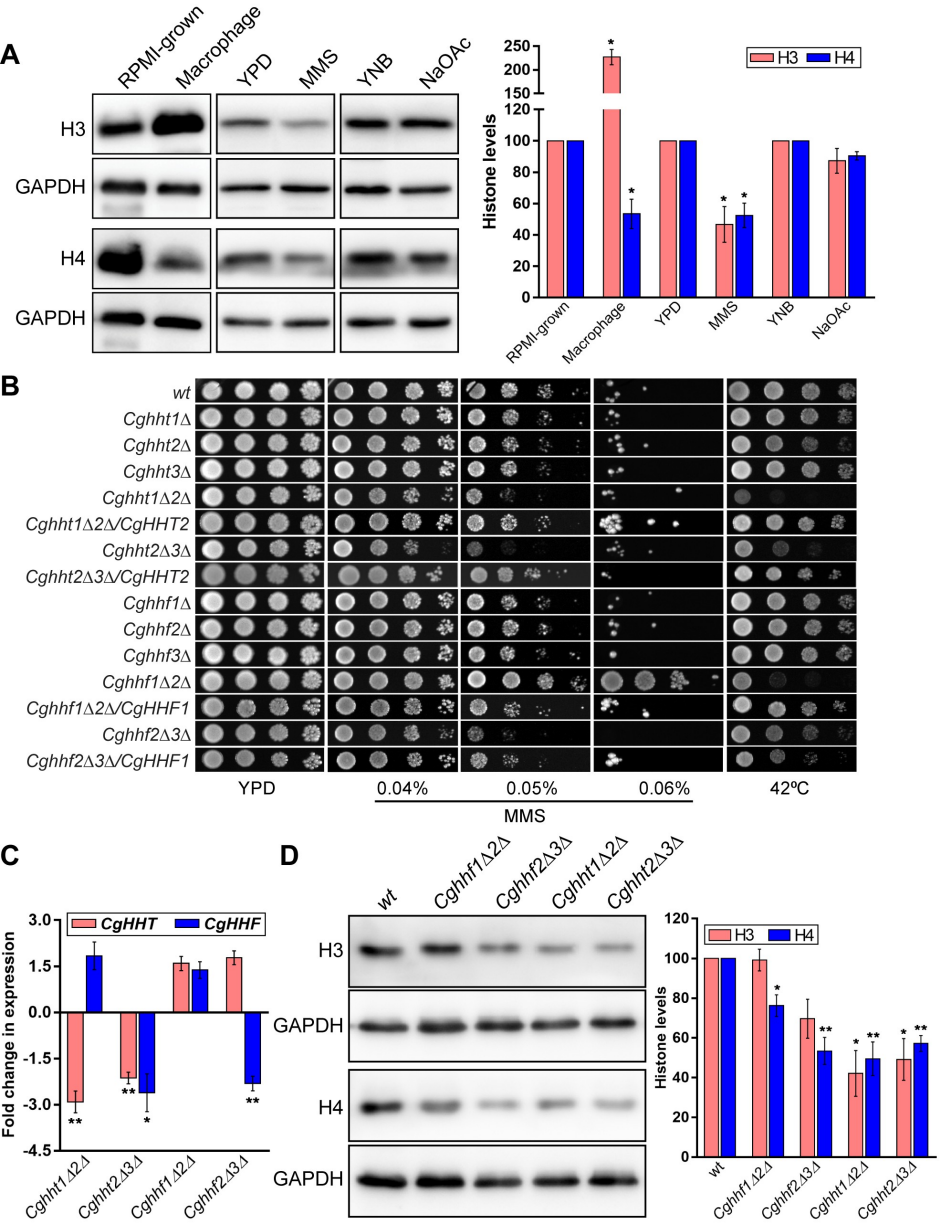
*C*. *glabrata* strains with reduced histone H3 and H4 gene dosage display differential MMS and thermal stress susceptibility. **A.** Representative immunoblots showing histone H3 and H4 levels in *wild-type* (*wt*) cells under indicated growth conditions. *C*. *glabrata* cells were grown in the YPD medium lacking or containing 0.03% MMS (MMS), YNB medium containing 2% dextrose (YNB) or 2% sodium acetate (NaOAc) or in the RPMI medium or infected to THP-1 macrophages (macrophages) for 6 h. For collection of macrophage-internalized cells, human THP-1 macrophages were infected with *C*. *glabrata* at 1:1 MoI (multiplicity of infection) and extracellular yeast cells were removed after 2 h. At 6 h post infection, macrophages were lysed in water and lysates were spun down to collect intracellular *C*. *glabrata* cells. Whole cell extracts were prepared by glass bead lysis of cells grown under all aforementioned conditions, and 50 μg protein were resolved on 15% SDS-PAGE and probed with anti-H3, anti-H4 and anti-GAPDH antibodies. CgGapdh was used as a loading control. Histone signal in each lane was quantified using the ImageJ software, and normalized against the CgGapdh signal. Data (mean ± SEM; *n =* 3) are plotted as a bar graph, and represent % change in histone signal in treated compared to control growth condition (taken as 100). *, *p* ≤ 0.05; paired two-tailed Student’s *t* test. **B.** Serial dilution spot assay to assess stress susceptibility of indicated *C*. *glabrata* strains. Cultures grown overnight in YPD medium were normalized to OD_600_ of 1.0, and diluted 10-fold serially in PBS. 3 μl of each dilution was spotted on the YPD medium lacking or containing different MMS concentrations, and plates were incubated at 30°C. For thermal stress, the YPD plate was incubated at 42⁰C. Images were captured after day 1 for 42°C, day 2 for YPD, 0.04% and 0.05% MMS, and day 3 for 0.06% MMS. **C.** qPCR-based measurement of histone H3 (*CgHHT*) and H4 (*CgHHF*) transcript levels. Total RNA (500 ng), extracted from log-phase *wt*, *Cghht1Δ2Δ*, *Cghht2Δ3Δ*, *Cghhf1Δ2Δ* and *Cghhf2Δ3Δ* strains using the acid phenol method, was used to set up the real-time quantitative reverse transcriptase-PCR reaction. Transcript levels were quantified using the 2-^ΔΔ^C_t_ method. Data (mean ± SEM, *n =* 3) were normalized against the *CgACT1* mRNA control, and represent fold change in *CgHHT* and *CgHHF* expression in histone mutants compared to *wt* strain. *, *p* ≤ 0.05, **, *p* ≤ 0.01; paired two-tailed Student’s *t* test. **D.** Representative immunoblots showing histone H3 and H4 levels in indicated *C*. *glabrata* strains. Whole-cell extracts were prepared by glass bead lysis of log-phase YPD-grown cultures. 50 μg protein were resolved on 15% SDS-PAGE and probed with anti-H3, anti-H4 and anti-GAPDH antibodies. CgGapdh was used as a loading control. For quantification, the intensity of individual bands in 4 independent Western blots was measured using the ImageJ densitometry software, and histone H3 and H4 signals were normalized to the corresponding CgGapdh signal. Data (mean ± SEM) represent % change in histone levels in mutants compared to *wt* (considered as 100), and are plotted as a bar graph on the right side of the blot image. *, *p* ≤ 0.05, **, *p* ≤ 0.01; paired two-tailed Student’s *t* test.

Macrophage-internalized *C*. *glabrata* cells have reduced histone H4 levels, reconfigured carbon metabolism and elevated phosphorylation at the Ser-129 residue of the histone H2A (referred to as γ-H2AX) [24]. The latter two responses are thought to be triggered by glucose limitation and/or presence of carbon sources other than glucose, and reactive oxygen species-induced DNA damage, respectively, in the macrophage internal milieu. Hence, to examine the effect of alternate carbon source and DNA damage on histone H4 levels, we performed Western blot analysis and observed a substantial (~50%) reduction in H4 levels in MMS-treated *C*. *glabrata* cells compared to untreated cells (Fig 1A), and upon macrophage internalization (Fig 1A), as reported previously [24]. Interestingly, MMS exposure also led to 2.1-fold decrease in H3 levels, though H3 levels were 2.3-fold elevated in macrophage-internalized *C*. *glabrata* cells (Fig 1A). Contrarily, H3 and H4 levels were similar between sodium acetate- and YNB medium-grown cells (Fig 1A). Altogether, the opposite effect of macrophage internal milieu and MMS treatment on H3 levels may reflect a cue-specific complex regulation of H3 levels, while reduced H4 levels in macrophage-ingested *C*. *glabrata* cells could reflect cellular response to the DNA damage.

Therefore, to assess the importance of histone H4 for the DNA damage response, we first identified three ORFs, *CAGL0C04136g*, *CAGL0H09834g* and *CAGL0M06677g*, in the *C*. *glabrata* genome that encode histone H4. *CAGL0C04136g* and *CAGL0M06677g* are syntenic to their *S*. *cerevisiae* counterparts *HHF1* and *HHF2*, respectively (S1A Fig). The *CAGL0H09834g* (*CgHHF3*) is the third additional H4-encoding ORF which is present with its cognate H3-encoding ORF *CAGL0H09856g* (*CgHHT3*) on the chromosome H in the genome (S1A Fig). Other two H3-encoding genes are *CAGL0C04114g* (*CgHHT1*) and *CAGL0M06655g* (*CgHHT2*), with genomic organization similar to that of *S*. *cerevisiae HHT1* and *HHT2* genes (S1A Fig). Notably, all three H3 and H4 ORFs in *C*. *glabrata* code for identical histone H3 (S1B Fig) and H4 (S1C Fig) proteins, respectively. To determine if presence of the additional copy of histones H3 and H4 in *C*. *glabrata*, as compared to *S*. *cerevisiae*, is a unique feature of pathogenic yeasts, we identified, through BLASTP, the number of histone H3 and H4 ORFs in the genomes of eleven fungal species including all yeasts of the *Nakaseomyces* Genus, to which *C*. *glabrata* belongs [29]. Of 6 known species of the *Nakaseomyces* genus, five yeasts (*Nakaseomyces delphensis*, *N*. *bacillisporus*, *C*. *nivariensis*, *C*. *bracarensis* and *C*. *glabrata*) has three H3- and H4-encoding ORFs, while the remaining species, *C*. *castelli* has two H3- and H4-encoding ORFs (S1D Fig). All four species of the *glabrata* group contain three H3- and H4-encoding ORFs (S1D Fig), indicating their close genetic relatedness. Further, since *C*. *castelli*, *N*. *delphensis* and *K*. *bacillicporus* are non-pathogenic, environmental species of the *Nakaseomyces* genus [29], the additional H3 and H4 ORF copy in *C*. *glabrata* is unlikely to be associated with its ability to infect the human host. In agreement with this, six other human pathogenic fungi contained one to three H3- and H4-encoding ORFs, with five fungi also containing unpaired H3 and H4 genes (S1D Fig). These data suggest that copies of the histone H3 and H4 genes may not be a good predictor of fungal virulence.

Next, we constructed strains, through homologous recombination, deleted for single, double or paired H3-H4 genes in *C*. *glabrata*. Despite multiple attempts, we were unable to create *Cghht1Δhht3Δ* and *Cghhf1Δhhf3Δ* mutants, suggesting that neither *CgHHT2* nor *CgHHF2* is sufficient for cell growth. Further, we checked growth profiles of histone H3- and H4-deleted mutants, and found all single (*Cghht1Δ*, *Cghht2Δ*, *Cghht3Δ*, *Cghhf1Δ*, *Cghhf2Δ* and *Cghhf3Δ*) mutants and one double mutant (*Cghhf1Δhhf2Δ*) to show growth similar to that of the *wild-type* (*wt*) strain (S2A Fig). In contrast, the remainder three double deletion strains, *Cghht1Δhht2Δ*, *Cghht2Δhht3Δ* and *Cghhf2Δhhf3Δ*, grew slowly, while the histone-paired deletion strain, *Cghht-hhf1Δ2Δ*, lacking two histone gene pairs (*CgHHT1-CgHHF1* and *CgHHT2-CgHHF2*), exhibited highly attenuated growth and significantly longer (38%) doubling time of 101.39 min, compared to that of *wt* cells (73.33 min) (S2A Fig).

*S*. *cerevisiae* histone H3 mutants have previously been reported to be temperature and DNA damage stress sensitive [30]. Therefore, we next conducted a comprehensive phenotypic analysis on *C*. *glabrata* histone H3 and H4 mutants, and found *Cghht1Δhht2Δ*, *Cghht2Δhht3Δ* and *Cghhf1Δhhf2Δ* mutants to be sensitive to thermal stress (Fig 1B). Additionally, *Cghht1Δhht2Δ* and *Cghht2Δhht3Δ*, and *Cghhf2Δhhf3Δ* mutants exhibited attenuated growth in the presence of the DNA damage-causing agent MMS, which was rescued by ectopic expression of *CgHHT2*, *CgHHT2* and *CgHHF1* genes, respectively (Fig 1B). Of note, single H3- and H4-deletion mutants showed no discernible phenotype (Fig 1B). While examining sensitivity to different genotoxic agents over a wide concentration range, we noticed that compared to *wt* cells, the *Cghhf1Δhhf2Δ* mutant exhibited better growth on higher concentration (0.06%) of MMS (Fig 1B). This enhanced growth was restored to *wt*-like growth upon ectopic expression of the *CgHHF1* gene from the *CgPDC1* promoter (Fig 1B), indicating that MMS resistance in the *Cghhf1Δhhf2Δ* mutant is owing to low dosage of the H4 gene. Consistently, functional complementation of MMS resistance in the *Cghhf1Δhhf2Δ* mutant was also observed upon expression of either of the three histone ORFs, *CgHHF1*, *CgHHF2* and *CgHHF3*, from their native promoters (S2B Fig). Since MMS resistance phenotype of the *Cghhf1Δhhf2Δ* mutant was unexpected, we further verified the effect of *CgHHF1* and *CgHHF2* gene loss on MMS susceptibility by generating four additional *Cghhf1Δhhf2Δ* deletion strains. All these independently generated *Cghhf1Δhhf2Δ* mutants showed sensitivity and resistance to elevated temperature and MMS, respectively, which was restored to *wt* levels upon *CgHHF1* expression (S2C Fig), thereby, corroborating the earlier finding that the loss of *CgHHF1* and *CgHHF2* genes conferred MMS resistance in *C*. *glabrata*.

Further, all histone mutants grew like *wt* strain in the medium containing oxidative stressor [hydrogen peroxide (H_2_O_2_)] and two other genotoxic agents [UV radiation and hydroxyurea (HU)], except for *Cghht1Δhht2Δ*, *Cghhf1Δhhf2Δ* and *Cghhf2Δhhtf3Δ* mutants (S3A Fig).While the *Cghht1Δhht2Δ* mutant displayed mild growth defect in the presence of HU, the *Cghhf1Δhhf2Δ* mutant displayed mild sensitivity to both UV and HU (S3A Fig). All three mutants (*Cghht1Δhht2Δ*, *Cghhf1Δhhf2Δ* and *Cghhf2Δhhtf3Δ*) were found to be susceptible to H_2_O_2_ (S3A Fig). Collectively, these data suggest that the *Cghhf1Δhhf2Δ* mutant is resistant specifically to the MMS-induced damage. Intriguingly, growth of *wt* cells overexpressing either *CgHHF1* or *CgHHT2* gene remained unperturbed at high temperature and in the MMS-containing medium (S3B Fig). Contrarily, compared to *wt* cells, the *Cghht1-hhf1Δhht2-hhf2Δ* mutant, that lacks two pairs of canonical histones H3 and H4, exhibited better growth in the presence of MMS, however, this MMS resistance was less than that in the *Cghhf1Δhhf2Δ* mutant (S3C Fig). Altogether, these data suggest that the low histone dosage differentially affects MMS resistance in *C*. *glabrata*, with loss of one set of two H4 genes leading to increased MMS susceptibility and the other set, consisting of canonical H4 genes, resulting in decreased MMS susceptibility. Although the precise molecular basis is yet to be elucidated, different amounts of H4 produced by the remaining H4 ORF is likely to contribute to this unexpected result.

### Histone H4 protein levels are modestly reduced in the *Cghhf1Δhhf2Δ* mutant

To delineate the basis underlying MMS resistance phenotype of the *Cghhf1Δhhf2Δ* mutant, we measured both H4 transcript and protein levels in H4-deleted strains. Compared to *wt* cells, we found a 2.5-fold and no reduction in H4 gene expression in *Cghhf2Δhhf3Δ* and *Cghhf1Δhhf2Δ* mutants, respectively (Fig 1C), indicating that H4 levels may not be drastically reduced in the *Cghhf1Δhhf2Δ* mutant. As a control, we also checked H3 transcript levels in both H4- and H3-deleted strains. H3 gene expression remained unaltered in both H4-deleted mutants (*Cghhf1Δhhf2Δ* and *Cghhf2Δhhf3Δ*), while H4 transcription was downregulated in the *Cghht2Δhht3Δ* mutant (Fig 1C). Expectedly, H3 transcript levels were lower in both H3-deleted strains (*Cghht1Δhht2Δ* and *Cghht2Δhht3Δ*) (Fig 1C). In addition to showing similar H4 transcript levels in *wt* and *Cghhf1Δhhf2Δ* strains, these qPCR data underscore the differential effects of H3 and H4 ORF loss on the transcription of the remainder H3 and H4 genes.

Further, Western blot analysis revealed about 25% and 50% reduction in H4 protein levels in the *Cghhf1Δhhf2Δ* and *Cghhf2Δhhf3Δ* mutants, respectively, compared to *wt* cells (Fig 1D). These results are not in total agreement with the transcriptional data, which showed similar H4 levels in *wt* and *Cghhf1Δhhf2Δ* strains, and may stem from posttranscriptional regulation of the *CgHHF3* gene product. A 30% decrease in H3 protein levels was also observed in the *Cghhf2Δhhf3Δ* mutant (Fig 1D). Contrarily, amounts of both H3 and H4 histones were ~ 2-fold lower in H3-deleted mutants, *Cghht1Δhht2Δ*, *Cghht2Δhht3Δ* (Fig 1D). These results are suggestive of an intricate regulation of histone protein levels in *C*. *glabrata*.

Altogether, four key findings emerge from aforementioned genetic and expression analyses. First, for both H3 and H4, at least one of the two genes (*CgHHT1*/*CgHHT3* and *CgHHF1*/*CgHHF3*) is required for cell growth. Second, *C*. *glabrata* cells lacking both canonical H3 and H4 gene pairs, *CgHHT1-HHF1* and *CgHHT2-HHF2*, are viable. Third, MMS resistance is specific to the absence of two H4 genes, *CgHHF1* and *CgHHF2*, and the simultaneous lack of their cognate H3 genes render cells more susceptible to MMS. The latter result could partly be due to restoration of the nucleosomal H3-H4 stoichiometry in the H3-H4 double paired-deletion strain. Finally, histone H4 levels modulate cellular response to MMS, with the *Cghhf2Δhhf3Δ* (~ 2-fold less H4) and *Cghhf1Δhhf2Δ* (~1.2-fold less H4) mutants showing increased and decreased MMS susceptibility, respectively. It is conceivable that a certain threshold amount of histone H4 is needed to survive MMS stress. An amount, lower than this, may hamper cellular ability to cope up with the MMS-induced DNA damage. Since MMS resistance phenotype of the *Cghhf1Δhhf2Δ* mutant was intriguing and unexpected, we chose this mutant to better understand the link between histone H4 and MMS stress survival, and the ‘low H4 gene dosage’ from hereon refers to the *Cghhf1Δhhf2Δ* mutant.

### MMS exposure substantially reduces H4 levels in the *Cghhf1Δhhf2Δ* mutant

**Fig 2:**
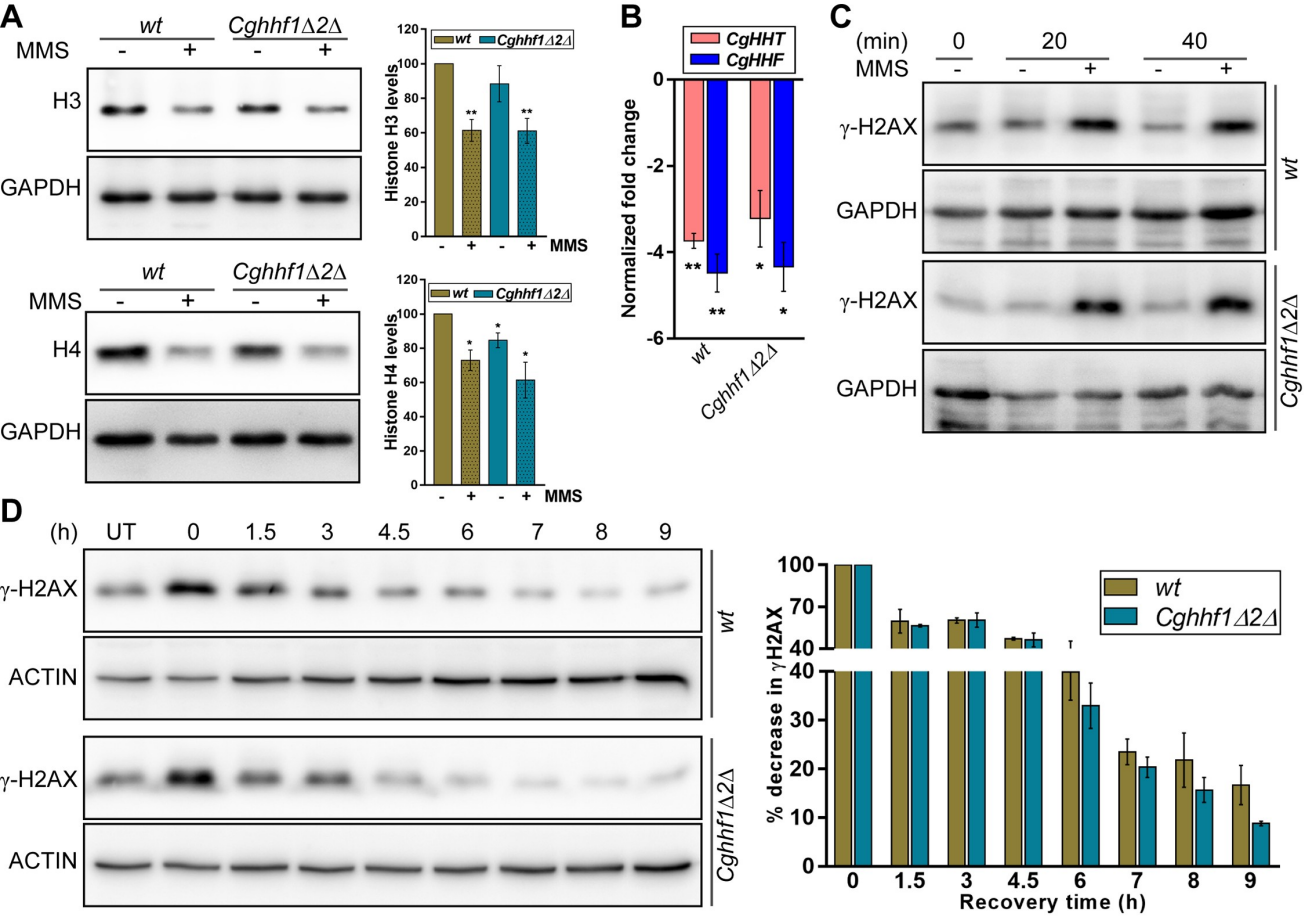
MMS exposure led to a substantial reduction in H3 and H4 levels. **A.** Representative immunoblots showing histone H3 and H4 levels in MMS-treated *wt* and *Cghhf1Δ2Δ* cells. Log-phase cultures were either left untreated or treated with 0.06% MMS for 45 min, whole cell lysates (50 μg) resolved on 15% SDS-PAGE and were probed with anti-H3, anti-H4 and anti-GAPDH antibodies. Data (mean ± SEM, *n =* 5) represent % change in histone levels compared to *wt* untreated samples (considered as 100), and are plotted as a bar graph on the right side of the blot image. *, *p* ≤ 0.05, **, *p* ≤ 0.01; paired two-tailed Student’s *t* test. **B.** qPCR-based measurement of histone H3 (*CgHHT*) and H4 (*CgHHF*) transcript levels. Indicated *C*. *glabrata* strains were either left untreated or treated with 0.06% MMS for 45 min. Data (mean ± SEM, *n =* 3) were normalized against the *CgACT1* mRNA control, and represent fold change in *CgHHT* and *CgHHF* expression in treated samples compared to untreated samples (taken as 1.0). *, *p* ≤ 0.05, **, *p* ≤ 0.01; paired two-tailed Student’s *t* test. **C.** A representative immunoblot showing γ-H2AX levels in *wt* and *Cghhf1Δ2Δ* cells. Log-phase cultures were grown either in the presence or absence of 0.06% MMS and cells were collected at 0, 20 and 40 min of MMS addition. 50 μg whole cell lysates were resolved on 15% SDS-PAGE and probed with anti-γH2AX and anti-GAPDH antibodies. **D.** A representative immunoblot showing recovery from the MMS-induced DNA damage. Log-phase *wt* and *Cghhf1Δ2Δ* cells were treated with 0.06% MMS for 45 min followed by cell collection and incubation in the fresh YPD medium lacking MMS. Cultures were harvested at indicated time points of recovery, whole cell lysates (50 μg) resolved on 15% SDS-PAGE and probed with anti-γH2AX and anti-actin antibodies. CgActin was used as a loading control. Data (mean ± SEM, *n =* 3) represent % reduction in γH2AX levels compared to 0 h samples (considered as 100), and are plotted as a bar graph on the right side of the blot image.

MMS-treated *wt* cells displayed a drastic reduction in H4 levels (Fig 1A), and low H4 gene dosage led to MMS resistance (Fig 1B), thereby, linking MMS response with H4 homeostasis. Hence, we next checked H4 levels in the MMS-resistant *Cghhf1Δhhf2Δ* mutant upon MMS exposure. We found that, similar to *wt* cells, H4 levels were drastically reduced in MMS-treated *Cghhf1Δhhf2Δ* cells (Fig 2A). Of note, MMS exposure also led to ~ 40% reduction in H3 protein amounts (Fig 2A). Consistently, ~ 3- to 5-fold lower expression of histone H3- and H4-encoding genes was observed in MMS-treated *wt* cells (Fig 2B), indicating a MMS-induced transcriptional and translational control of H3 and H4 gene expression. Importantly, MMS exposure also led to similar reduction in H3 and H4 transcript levels in the *Cghhf1Δhhf2Δ* mutant (Fig 2B), as well as in *Cghhf2Δhhf3Δ*, *Cghht1Δhht2Δ* and *Cghht2Δhht3Δ* mutants (S3D Fig), underscoring that *CgHHT1* and *HHT3*, and *CgHHF1* and *HHF3* genes, are repressed in response to MMS.

### MMS-treated *wt* and *Cghhf1Δhhf2Δ* cells display higher γ-H2AX levels

MMS methylates DNA at 7-deoxyguanine and 3-deoxyadenine bases which predominantly results in replication fork stalling [31]. However, double-strand breaks in DNA can indirectly be generated during repair of MMS-modified bases [32]. Since γ-H2AX is involved in repair of the double-strand breaks [33], we next checked γ-H2AX levels in MMS-treated *wt* and *Cghhf1Δhhf2Δ* cells. A 20 min MMS treatment led to ~ 2 to 3-fold increase in γ-H2AX amounts in both *wt* and *Cghhf1Δhhf2Δ* mutant cells (Fig 2C). This result indicates that sensing and mounting of an appropriate response to the MMS-induced DNA damage is similar in *wt* and *Cghhf1Δhhf2Δ* cells. Conceivably, MMS resistance in the *Cghhf1Δhhf2Δ* mutant could then be due to faster repair of the damaged DNA, which can be monitored by reduction in γ-H2AX levels. Hence, we measured γ-H2AX levels at different time points after MMS removal, and observed a marginally faster reduction in γ-H2AX levels in *Cghhf1Δhhf2Δ* cells from 6 h onwards, compared to *wt* cells (Fig 2D). Consequently, the γ-H2AX signal in MMS-treated *Cghhf1Δhhf2Δ* cells was about 2-fold lower than that in MMS-treated *wt* cells after 9 h of recovery (Fig 2D), indicating that the mutant may recover slightly faster from the MMS-induced DNA damage. Consistent with this result, 40% of the *Cghhf1Δhhf2Δ* mutant population survived the 4 h MMS treatment, compared to 12% of the *wt* population (S3E Fig). Altogether, these data indicate that the *CgHHF1* and *CgHHF2* gene loss confers a modest survival advantage under MMS stress, which may in part be owing to the agile DNA damage repair system.

### Homologous recombination pathway is more efficient in the *Cghhf1Δhhf2Δ* mutant

In view of a faster recovery of *Cghhf1Δhhf2Δ* cells from the DNA damage, we set out to delineate the DNA repair process responsible for this effect. Two main pathways for DNA damage repair are homologous recombination (HR) and non-homologous end joining (NHEJ) recombination. Although *C*. *glabrata* contains both pathways, it has higher NHEJ than *S*. *cerevisiae* [34,35]. To characterize repair mechanisms of the MMS-induced DNA damage in *wt* and *Cghhf1Δhhf2Δ* cells, we performed two experiments. First, we measured the efficiency of HR and NHEJ through linear fragment recombination and plasmid circularization assay, respectively. Second, we generated *CgyKU80* and *CgRAD52* deletions in the *Cghhf1Δhhf2Δ* mutant background, with CgyKu80 and CgRad52 being the core constituents of NHEJ and HR pathway, respectively. Cgyku80 is required for telomere length maintenance and NHEJ-mediated DNA repair in *C*. *glabrata* [36,37]. CgRad52 in *C*. *glabrata* is uncharacterized, however, its ortholog in *S*. *cerevisiae* is involved in repair of the DNA double-strand breaks via strand exchange stimulation [38]. CgRad52 showed 60% similarity with ScRad52, and contained the conserved RAD52_Rad22 domain at its N-terminus and two nuclear localization sequences (S4 Fig).

**Fig 3:**
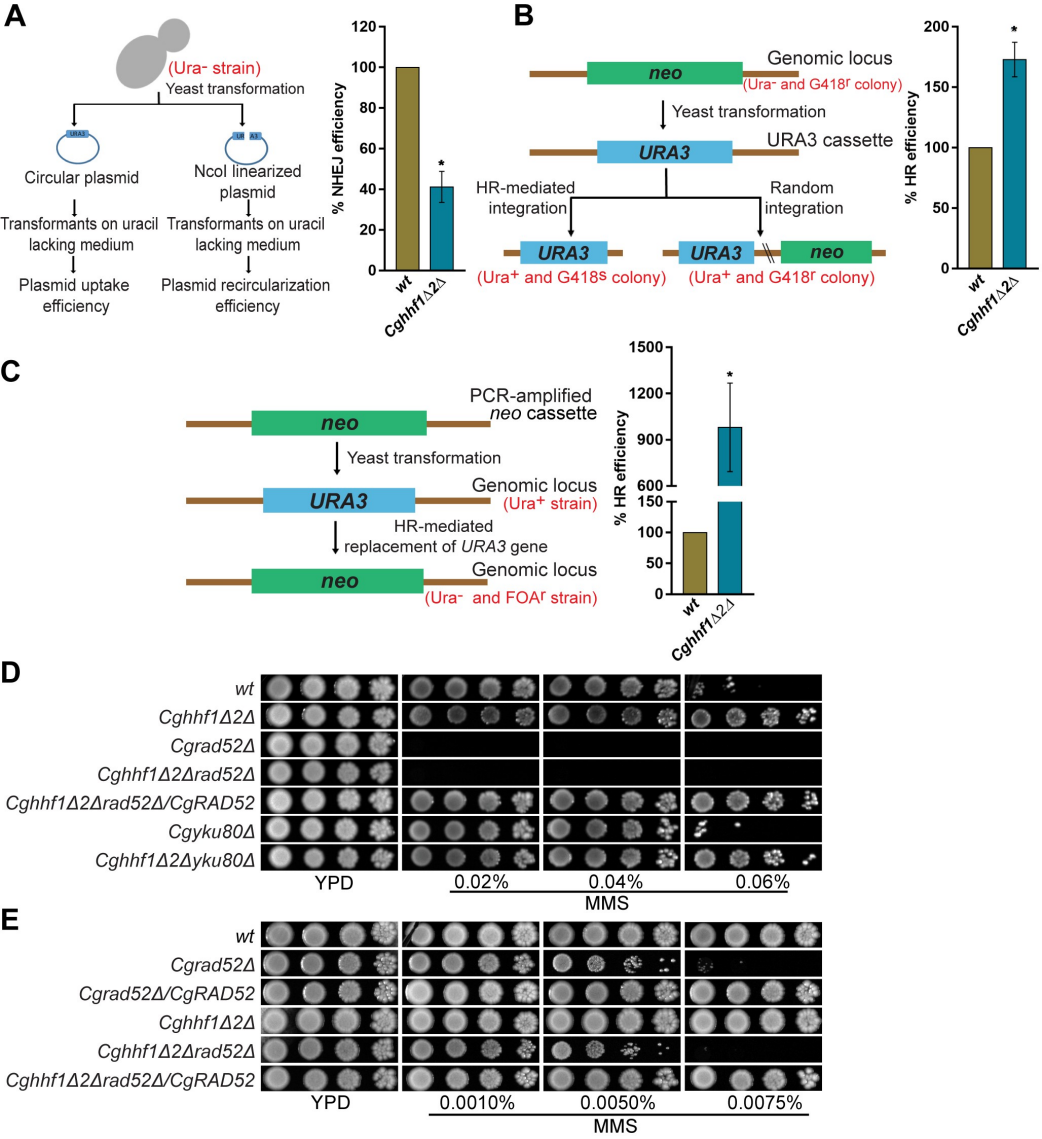
Homologous recombination pathway is required to survive MMS stress. **A.** Quantification of the non-homologous end joining (NHEJ) efficiency. Log-phase, uracil auxotroph *wt* and *Cghhf1Δ2Δ* cells were transformed with the circular plasmid pRK74 (100 ng) or the NcoI-linearized pRK74 plasmid (250 ng), and the number of uracil prototroph transformants obtained was counted. The percentage efficiency of plasmid re-circularization was calculated using the formula [(number of transformants obtained with linear plasmid/number of transformants obtained with circular plasmid) x 100] and plotted. The NHEJ efficiency in *wt* was considered as 100. Data represent mean ± SEM (*n =* 3). *, p ≤ 0.05; paired two-tailed Student’s t test. Schematic representation of the NHEJ assay is shown on the left side of the bar graph. **B.** Quantification of the homologous recombination (HR) efficiency. Log-phase *wt* and *Cghhf1Δ2Δ* cells containing *neo* gene in place of *CgURA3* were transformed with the PCR amplified *CgURA3* gene (500 ng) carrying 750 and 550 bp of 5’ and 3’ UTRs, respectively. The number of uracil prototroph transformants obtained was counted and checked for G418 (150 mg/ml) resistance. The percentage HR efficiency was calculated by dividing the total number of uracil prototroph and G418 sensitive colonies by the total number of uracil prototroph transformants, and multiplying this number by 100, and plotted. The HR efficiency in *wt* was considered as 100. Data represent mean ± SEM (*n =* 3). *, p ≤ 0.05; paired two-tailed Student’s t test. Schematic representation of the HR assay is shown on the left side of the graph. **C.** FOA resistance-based HR efficiency measurement. Log-phase *wt* and *Cghhf1Δhhf2Δ* strains (uracil prototrophs) were transformed with a PCR amplified 5’*CgURA3* UTR-*neo*-3’*CgURA3* UTR cassette (500 ng), and plated on CAA medium containing FOA (1.1 mg/ml). The number of FOA resistant colonies obtained for each strain was counted. All strains were also transformed with a circular plasmid expressing *hph1* gene, selected for hygromycin (500 μg/ml) resistance and the number of hygromycin resistant transformants was determined. The percentage HR efficiency was calculated by dividing the total number of FOA resistant colonies by the total number of hygromycin-resistant transformants, and multiplying this number by 100. The HR efficiency in *wt* was considered as 100. Data represent mean ± SEM (*n =* 3). *, p ≤ 0.05; paired two-tailed Student’s t test. Schematic representation of the HR assay is shown on the left side of the graph. **D-E.** Serial dilution spot assay showing susceptibility of indicated *C*. *glabrata* strains on high **(D)** and low **(E)** concentrations of MMS.

Compared to *wt* cells, we found 2.5-fold lower and 1.7-fold higher rate of NHEJ and HR, respectively, in the *Cghhf1Δhhf2Δ* mutant (Fig 3A and 3B). Since this HR efficiency was calculated by dividing the number of homologous recombinants by the sum total of homologous and non-homologous recombinants, the higher HR efficiency in the *Cghhf1Δhhf2Δ* mutant could be due to diminished NHEJ efficiency. Therefore, to rule out the effect of reduced NHEJ rate on HR efficiency, we measured HR efficiency by another assay, wherein resistance to FOA (5-Fluoroorotic acid) was scored as a direct read-out of the HR efficiency. This analysis revealed 10-fold higher HR efficiency in the *Cghhf1Δhhf2Δ* mutant, compared to *wt* cells (Fig 3C), thereby, corroborating the earlier finding that the *Cghhf1Δhhf2Δ* mutant probably has more efficient HR machinery.

Further, through mutant analysis, we observed a complete reversal of MMS resistance phenotype of the *Cghhf1Δhhf2Δ* mutant, upon deletion of the *CgRAD52* gene (Fig 3D), as both *Cgrad52Δ* and *Cghhf1Δhhf2Δrad52Δ* mutants displayed exquisite MMS sensitivity (Fig 3E). Although these data indicate a link between histone H4 and CgRad52, the possibility of H4 and CgRad52 acting independently in MMS response can not be precluded. Notably, *CgyKU80* disruption had no effect on DNA damage susceptibility of *wt* or *Cghhf1Δhhf2Δ* cells (Fig 3D). Altogether, these data indicate that MMS-induced DNA damage in *C*. *glabrata* is predominantly repaired by HR, and that, MMS resistance in the *Cghhf1Δhhf2Δ* mutant is dependent upon a functional HR machinery.

### RNA-Seq analysis of the *Cghhf1Δhhf2Δ* mutant revealed elevated expression of DNA repair and adhesin genes

**Fig 4:**
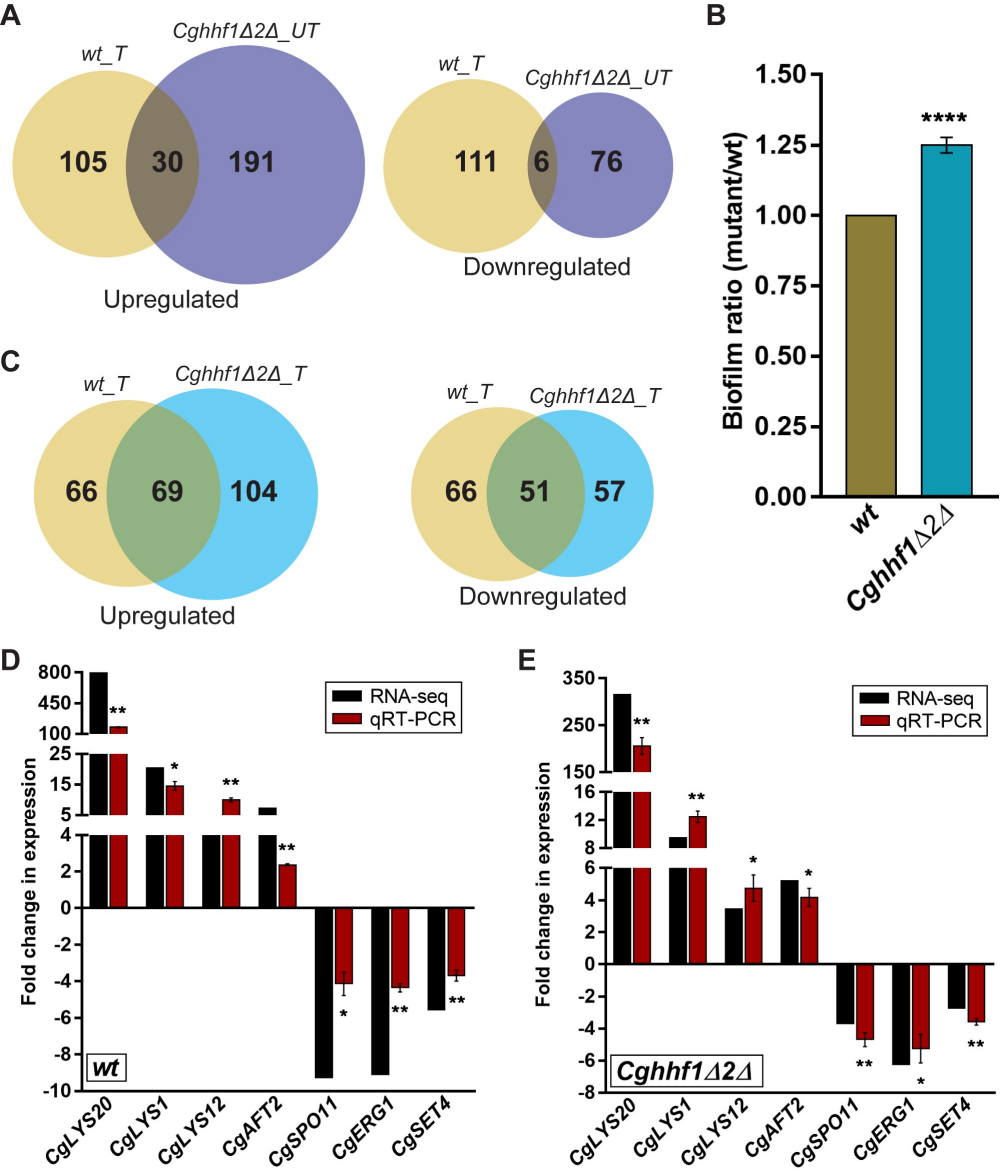
RNA-Seq-based transcriptome profiling in response to low H4 gene dosage and MMS. RNA was extracted from log-phase cultures of untreated and MMS-treated (0.06% MMS; 45 min treatment) *wt* and *Cghhf1Δ2Δ* strains, libraries prepared using the Truseq RNA library prep kit, and 100 bp paired-end sequencing was performed on Illumina Hiseq 2500. Differentially expressed genes, that showed ≥ 2-fold change in expression and a *p* value of ≤ 0.05, were identified using the DESeq program. **A.** Venn diagrams illustrating overlap in differentially expressed genes between untreated *Cghhf1Δ2Δ* mutant (*Cghhf1Δ2Δ_UT*) and MMS-treated *wt* (*wt_T*) cells. **B.** Biofilm formation assay. *wt* and *Cghhf1Δhhf2Δ* strains were grown in RPMI medium containing 10% FBS in a 24-well polystyrene plate. After 48 h incubation, the biofilm, formed by yeast cells on polystyrene, was stained with 0.4% crystal violet for 45 min, followed by three PBS washes. Post destaining with 95% ethanol, biofilm mass was measured by monitoring the absorbance at 595 nm. Data (mean ± SEM; *n =* 5) represent biofilm ratio, which was calculated by dividing the absorbance units of *Cghhf1Δhhf2Δ* mutant by those of the *wt* strain (considered as 1.0). ****, *p* ≤ 0.001; paired two-tailed Student’s t test. **C.** Venn diagrams illustrating overlap in differentially expressed genes between MMS-treated cells of *wt* (*wt_T*) and *Cghhf1Δ2Δ* (*Cghhf1Δ2Δ_T*) strains. **D-E.** qPCR verification of the RNA-Seq data. Log-phase *wt* and *Cghhf1Δ2Δ* cells were grown in the absence and presence of 0.06% MMS for 45 min, RNA extracted and qPCR was performed. Transcript levels of indicated genes (four upregulated and three downregulated genes in the RNA-Seq experiment) were quantified by 2-^ΔΔ^C_t_ method. Data (mean ± SEM, *n =* 3) were normalized against the *CgACT1* mRNA control and represent fold change in expression in MMS-treated samples compared to untreated *wt* **(D)** and *Cghhf1Δ2Δ* **(E)** samples. *, *p* ≤ 0.05, **, *p* ≤ 0.01; paired two-tailed Student’s *t* test.

As changes in the stoichiometry of core histones impact transcription in *S*. *cerevisiae* [39], we reasoned that the *CgHHF1* and *CgHHF2* gene loss may trigger induction of HR genes, rendering the HR pathway more efficient in the *Cghhf1Δhhf2Δ* mutant. Hence, we next profiled the whole transcriptome of log-phase *wt* and *Cghhf1Δhhf2Δ* cells under both normal and MMS treatment conditions using the RNA-Sequencing approach. Transcriptome comparison of log-phase *wt* and *Cghhf1Δhhf2Δ* cells revealed that *CgHHF1* and *CgHHF2* gene loss led to altered transcription of 303 genes, with 221 and 82 showing induction and repression, respectively, in the *Cghhf1Δhhf2Δ* mutant (Fig 4A; S1 Table), precluding an ecumenical increase in transcript abundance due to reduced H4 gene dosage. Of note, H3 and H4 depletion in *S*. *cerevisiae* led to elevated expression of 38% [40] and 15% [41] of total genes, respectively. In contrast, low H4 dosage in *C*. *glabrata* resulted in increased and decreased expression of 4.2% and 1.6% total genes, respectively (S1 Table). The *C*. *glabrata* transcriptome data also suggest that the nucleosomal architecture may not be drastically altered globally in the *Cghhf1Δhhf2Δ* mutant. Further, compared to *wt* chromatin, chromatin in the *Cghhf1Δhhf2Δ* mutant displayed slightly enhanced sensitivity to the micrococcal nuclease (MNase) digestion (S5 Fig). This is in contrary to the MNase-resistant chromatin of mid-phase macrophage-internalized *C*. *glabrata* cells, which also contained reduced H4 levels [24]. This discrepancy could in part be due to specific post-translational modifications, as modifications of histone H3 and H4 proteins, that represent transcriptionally silent (heterochromatin) and active (euchromatin) chromatin, were increased and decreased, respectively, in intracellular *C*. *glabrata* cells, after 6–12 h infection to macrophages [24]. Altogether, these data indicate that the histone H4 levels may not be a sole determinant of the chromatin architecture. Of note, depletion of the linker histone H1 in mouse embryonic stem cells has previously been associated with a more open chromatin structure [42].

Strikingly, *CgHHF1* and *CgHHF2* loss resulted in the enrichment of ‘maturation of large and small subunit-rRNA from tricistronic rRNA transcript’ and ‘ribosomal biogenesis’ biological processes (BPs) in upregulated, and ‘amino acid biosynthesis’ and ‘one-carbon metabolic processes’ in downregulated genes (S2 Table). Further, expression of 7 DNA repair genes (*CgTEL1*, *CgNTG1*, *CgOGG1*, *CgPSF2*, *CgRTF1*, *CgHIM1* and *CgHRQ1*) was found to be upregulated in *Cghhf1Δhhf2Δ* mutant, compared to *wt* cells (S1 Table), reflecting altered DNA repair mechanism/s in the mutant.

H4 depletion in *S*. *cerevisiae* has been reported to result in de-repression of the telomere-proximal genes [41]. Since several adhesin genes in *C*. *glabrata* are encoded at the subtelomeric loci [43], we studied the effect of low H4 dosage on expression of genes located near the telomeres. For this, we extracted all ORFs located within a 40 kb distance from the ends of all 13 *C*. *glabrata* chromosomes (www.candidagenome.org), and examined the expression of this set of 314 genes in the *Cghhf1Δhhf2Δ* mutant. We found only 1.6% and 5.7% of these subtelomeric genes to be downregulated and upregulated, respectively, in the *Cghhf1Δhhf2Δ* mutant (S3 Table). Furthermore, of 66 adhesin-encoding genes present in *C*. *glabrata* [44], four genes were upregulated, while one adhesin gene was downregulated in the *Cghhf1Δhhf2Δ* mutant (S1 Table). The induced adhesin gene set constituted 9% of subtelomeric adhesin genes [44], indicating a minor role for the histone H4 in transcriptional silencing of the subtelomeric genes in *C*. *glabrata*.

Next, to determine the physiological relevance of elevated adhesin gene expression in the *Cghhf1Δhhf2Δ* mutant, we measured the ability of *wt* and *Cghhf1Δhhf2Δ* cells to form biofilm on polystyrene, and found it to be 25% higher than *wt* cells (Fig 4B), indicating that the histone H4 dosage modulates biofilm formation capacity of *C*. *glabrata* cells.

Further, MMS exposure led to differential expression of 252 genes, with 135 and 117 genes showing upregulation and downregulation, respectively, in *wt* cells (Fig 4C; S4 Table). Of note, 36 genes were common between MMS-treated and *CgHHF1-CgHHF2*-deleted *C*. *glabrata* cells (Fig 4A; S1 and S4 Tables). The number of differentially expressed genes (DEGs) in MMS-treated *Cghhf1Δhhf2Δ* cells was 281, with 173 up- and 108-downregulated genes (Fig 4C; S5 Table). The overall transcriptional profiles of MMS-treated *wt* and *Cghhf1Δhhf2Δ* cells were not vastly different, as they shared a common set of 120 DEGs (Fig 4C). Next, we verified the RNA-Seq data for 4 induced and 3 repressed genes in MMS-treated cells of both *wt* (Fig 4D) and *Cghhf1Δhhf2Δ* strains (Fig 4E) by qPCR and found good correlation (Fig 4D and 4E).

GO enrichment analysis of DEGs in MMS-treated *wt* cells identified ten significant BPs, with two (transmembrane transport and invasive growth in response to glucose limitation) in upregulated, and eight including aerobic respiration and ergosterol biosynthetic process, in downregulated genes (S6 Table). The histidine and lysine biosynthetic process, and mitochondrial electron transport, ribosomal large subunit assembly and rRNA processing BPs were enriched in induced and repressed gene sets, respectively, in MMS-treated *Cghhf1Δhhf2Δ* cells (S7 Table). Altogether, these data indicate a widespread transcriptional response of *C*. *glabrata* to the MMS insult, which probably reflects more the metabolic response to DNA damage than the DNA repair response. An important link between metabolism and DNA repair processes has recently been identified [45], the expansive response of *C*. *glabrata* to MMS is, thus, not unprecedented.

### Mass spectrometry analysis revealed lower number of interactions for histone H4 upon MMS treatment

Since the transcriptional profiling of *Cghhf1Δhhf2Δ* mutant did not yield insights into the mechanistic link between H4 dosage and HR-dependent repair of MMS-induced DNA damage, we sought to identify proteins that interact with H4 under normal (CAA medium growth) and MMS-treated conditions. Furthermore, to determine interactors of the chromatin-associated H4, we performed the histone H3 interactome analysis, with the rationale that the chromatin-associated H3 and H4 are likely to bind to the same set of proteins. For this, we tagged H3 and H4 proteins with the triple SFB epitope at their C-termini, and verified the expression and functionality of the tagged proteins by Western blot (S6A Fig) and respective mutant complementation (S6B Fig) analyses, respectively.

**Fig 5:**
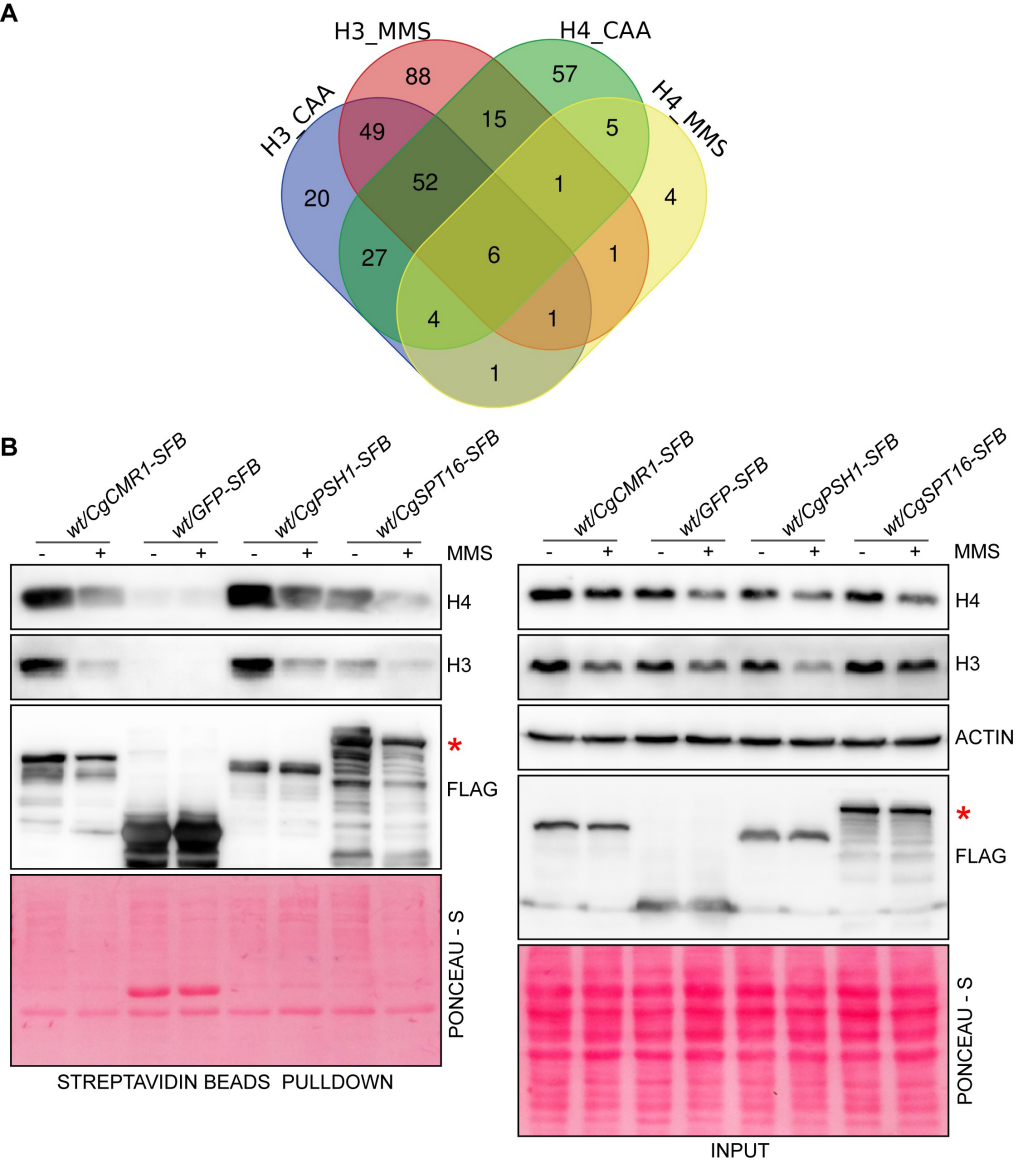
Mass spectrometry analysis revealed a smaller H4 interactome upon MMS treatment. **A.** Venn diagram illustrating overlap in interacting proteins of histones H3 and H4 under normal (growth in CAA) and MMS treatment conditions. Venn diagram was made using the freely available web tool at URL http://bioinformatics.psb.ugent.be/webtools/Venn/. **B.** Validation of the interaction of CgCmr1, CgPsh1 and CgSpt16 with histones H3 and H4. Log-phase *wt* strain expressing CgCmr1-SFB, GFP-SFB, CgPsh1-SFB or CgSpt16-SFB was grown in the absence or presence of 0.06% MMS for 3 h. Whole cell extracts were prepared and incubated with streptavidin beads. After 2 h incubation, beads were boiled in 2X SDS dye and resolved on 12% SDS-PAGE along with whole cell extracts (80 μg; Input). The red asterisk marks the CgSpt16-SFB protein band.

Next, we subjected the whole cell extracts of untreated and MMS-treated *Cghht1Δhht2Δ* and *Cghhf1Δhhf2Δ* cells, expressing *CgHHT1-SFB* and *CgHHF3-SFB*, respectively, to two-step affinity purification, followed by MS analysis. The MS analysis identified 167 and 23 proteins to interact with H4 under normal and MMS-treatment conditions, respectively (S8 Table; Fig 5A), indicating a substantial reduction (86%) in H4 interactions upon MMS treatment. Unexpectedly, the number of H3 interactors in MMS-treated cells was 33% higher, with 160 and 213 proteins interacting with H3 under normal and MMS-treatment conditions, respectively (Fig 5A; S9 Table). A set of 108 H3- and 16 H4-interacting proteins was common between CAA-grown and MMS-treated *C*. *glabrata* cells. Furthermore, H3 and H4 interacted with the same set of 89 and 9 proteins under normal and MMS treatment conditions, respectively (Fig 5A). A wide range of processes including ribosomal large subunit biogenesis and ATP-dependent chromatin remodelling were enriched in the FungiFun-GO analysis of 89 proteins, indicating a pivotal role for histones H3 and H4 in translation and chromatin organization processes (S10 Table). The analysis of MMS-specific 9 H3- and H4-interactors revealed enrichment of histone acetylation and chromatin silencing BPs (S10 Table).

Notably, the ‘cell adhesion’ process was uniquely enriched in the CAA-H4 interactome analysis, which may have implications for the pathogenesis of *C*. *glabrata* (S11 Table). Moreover, despite the large number of MMS-specific H3 interactors (105), the FungiFun analysis revealed enrichment of only three BPs, nucleosome disassembly, pre-replicative complex assembly and mitochondrial citrate transport (S12 Table). Contrarily, negative regulation of histone H3-K9 methylation, nucleotide-excision repair, and mismatch repair BPs showed enrichment in the analysis of 7 MMS-specific H4 interactor analysis (S11 Table), pointing towards a more prominent role for the histone H4 in repair of the MMS-induced DNA damage. Lastly, the fewer number of H4 interactors in MMS-treated *C*. *glabrata* cells could solely not be due to decreased H4 levels, as MMS induced similar reduction in H3 and H4 amounts (Fig 2A), and may reflect a distinct feature of the DNA damage response regulatory network in *C*. *glabrata*. The enriched terms for all GO BPs for histone H4 and H3 interactors are listed in S11 and S12 Tables, respectively.

Comparison of the *C*. *glabrata* H3 and H4 interactome with the published H3 and H4 interacting protein data for *S*. *cerevisiae*, available on the BIOGRID database, revealed 43% and 40% overlap with known H3 and histone H4 interactors, respectively (S6C Fig). Based on this analysis, we have identified 151 and 103 novel interacting proteins of H3 and H4, respectively.

To validate the H3 and H4 interactome data, we selected three proteins, CgCmr1, CgSpt16 and CgPsh1, based on their probable role in histone homeostasis. These proteins are all uncharacterized in *C*. *glabrata*, and showed interaction with both H3 and H4 under normal and MMS conditions in the pull-down experiment. The *S*. *cerevisiae* ortholog of CgSpt16 binds to histones and is a subunit of the nucleosomal organization FACT (FAcilitates Chromatin Transcription) complex [46,47]. Psh1 in *S*. *cerevisiae* is an E3 ubiquitin ligase involved in localization and degradation of the histone H3 variant Cse4 [48]. Cmr1 in *S*. *cerevisiae* is a DNA-binding protein that plays an important role in protein quality control by regulating the stability of repair and replication proteins [49,50]. It is also required for recovery after replication stress, and genome integrity maintenance [50,51]. We tagged CgCmr1, CgSpt16 and CgPsh1 proteins with the SFB epitope, and verified the interaction of tagged proteins with histones H3 and H4 by affinity purification followed by immunoblot analysis. GFP-SFB (~ 40 kDa), CgCmr1-SFB (~ 85 kDa), CgSpt16-SFB (~ 130 kDa) and CgPsh1-SFB (~70 kDa) proteins were present in both input and pull-down samples (Fig 5B). Further, histones H3 and H4 were found in input samples of all strains but in the pull-down samples of only CgCmr1, CgSpt16 and CgPsh1-expressing strains (Fig 5B), indicating a specific interaction of histones H3 and H4 with CgCmr1, CgSpt16 and CgPsh1 proteins. Notably, all three proteins interacted with H3 and H4 under both normal and MMS-treatment conditions (Fig 5B), thereby, corroborating the proteomic data.

### CgCmr1 is required for maintenance of histone H4 levels and homologous recombination

**Fig 6:**
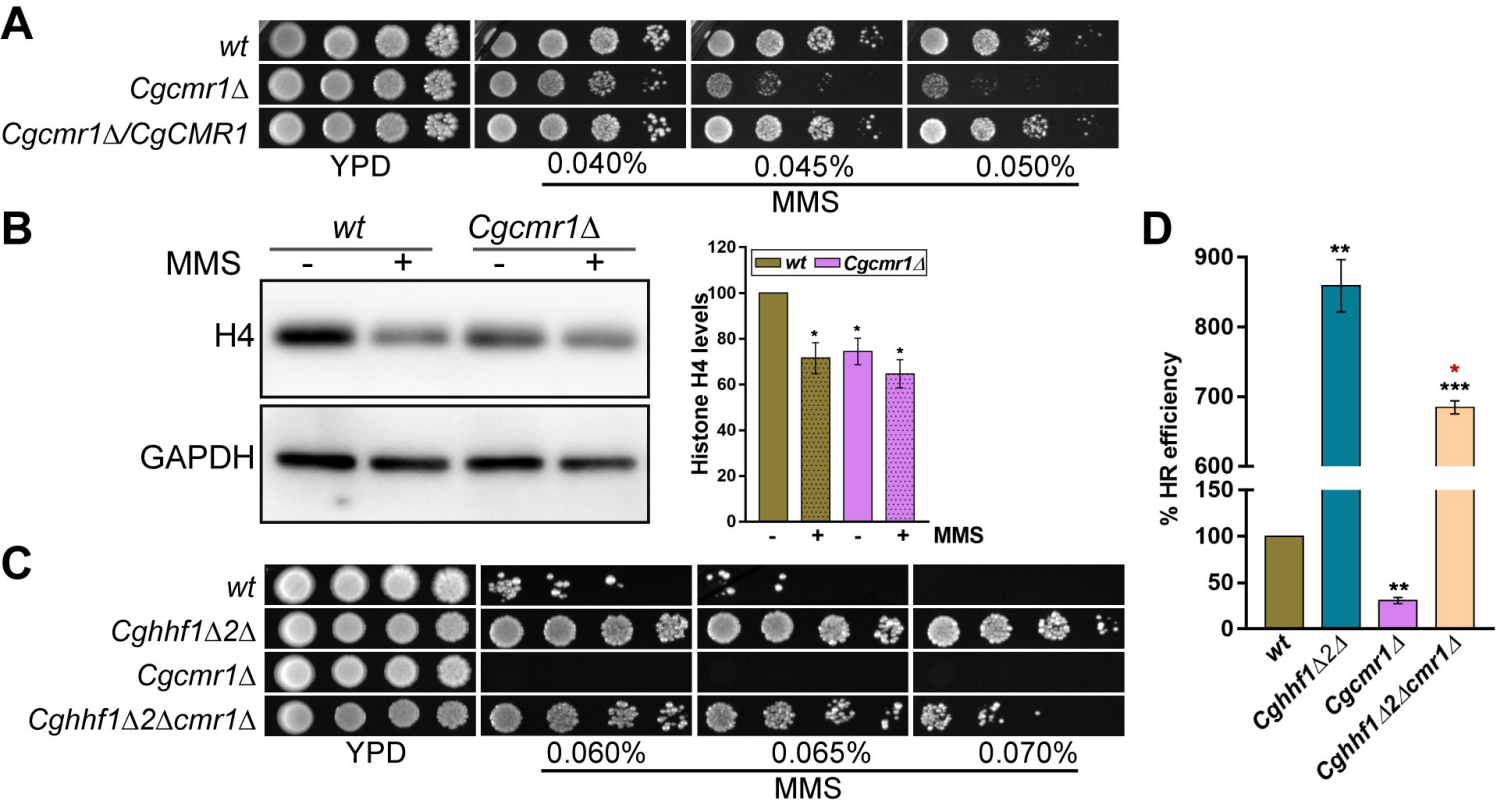
CgCmr1 is required for HR and MMS stress survival. **A.** Serial dilution spot assay showing elevated MMS susceptibility of the *Cgcmr1Δ* mutant, which was rescued upon ectopic expression of the *CgCMR1* gene. **B.** A representative immunoblot showing H4 levels in whole cell extracts (50 μg) of *wt* and *Cgcmr1Δ* strains, which were either treated with 0.06% MMS for 45 min or left untreated. % change in H4 levels (mean ± SEM; n = 4), compared to *wt* untreated samples (considered as 100), was quantified using the ImageJ software, and presented as a bar graph on the right side of the blot image. *, *p* ≤ 0.05; paired two-tailed Student’s t test. **C.** Serial dilution spot assay showing MMS susceptibility of indicated *C*. *glabrata* strains. **D.** A bar graph showing % HR efficiency, as determined by FOA resistance-based assay, in indicated *C*. *glabrata* strains. Black and red asterisks indicate statistically significant differences between *wt* and mutants, and *Cghhf1Δ2Δ* and *Cghhf1Δ2Δcmr1Δ* mutants, respectively. Data represent mean ± SEM (*n =* 3). **, *p* ≤ 0.01; ***, *p* ≤ 0.005; paired two-tailed Student’s *t* test. *, *p* ≤ 0.05; unpaired two-tailed Student’s t test.

During protein interaction validation analysis, we noticed that histone H4 levels were not decreased in *wt* cells overexpressing CgCmr1-SFB (Fig 5B). Since CgCmr1 is an uncharacterized protein in *C*. *glabrata*, we performed BLASTP analysis and found that CgCmr1 shares 67% identity with *S*. *cerevisiae* Cmr1 protein, and contains 6 units of WD-40 repeats (protein-protein interaction motifs) (S7A Fig). To examine if CgCmr1 is involved in maintenance of H4 levels upon DNA damage, we performed three experiments. First, we generated the *Cgcmr1Δ* mutant and found it to be sensitive to MMS (Fig 6A), implicating CgCmr1 in MMS stress survival. Second, we checked histone H4 levels in the *Cgcmr1Δ* mutant under normal and MMS-treated conditions. H4 levels were lower in the CAA-grown mutant (Fig 6B), indicating a requirement for CgCmr1 in regulation of H4 protein levels. Importantly, the H4 transcript levels in the *Cgcmr1Δ* mutant were similar to *wt* cells (S7B Fig). Moreover, MMS exposure, like in *wt* cells, led to diminished H4 transcript (S7B Fig) and protein levels (Fig 6B) in the *Cgcmr1Δ* mutant. Third, we disrupted the *CgCMR1* gene in the *Cghhf1Δhhf2Δ* mutant background, and found the triple knock-out strain to be more susceptible to MMS compared to the *Cghhf1Δhhf2Δ* mutant (Fig 6C). The *CgCMR1* loss-mediated partial rescue of MMS resistance in the *Cghhf1Δhhf2Δ* mutant reflects that the lack of CgCmr1 partially offsets the positive impact of low histone dosage on MMS stress survival. It is possible that CgCmr1-H4 interaction is either required to maintain H4 levels at a critical value, that permits *C*. *glabrata* cells survive in the presence of MMS, or for the recruitment/stabilization of HR factors to initiate repair of the damaged DNA.

After establishing that CgCmr1 and histone H4 interact physically as well as genetically, we next probed the significance of this interaction in MMS resistance conferred by low H4 gene dosage. Since key component of the HR pathway CgRad52 is required for survival of MMS stress (Fig 3D), and the MMS-resistant *Cghhf1Δhhf2* mutant showed higher HR efficiency (Fig 3B and 3C), we checked the efficiency of homologous recombination in the *Cgcmr1Δ* and triple deletion *Cghhf1Δhhf2Δcmr1Δ* strains. We found that the *Cgcmr1Δ* mutant was deficient in HR, as it showed 3.3-fold lower HR efficiency (Fig 6D). Contrarily, the HR efficiency in the *Cghhf1Δhhf2Δcmr1Δ* mutant was 6.8-fold higher and 1.25-fold lower, compared to that in *wt* and *Cghhf1Δhhf2Δ* mutant, respectively (Fig 6D). These results are consistent with the partial reversal of MMS resistance observed in the triple deletion strain (Fig 6C). Altogether, these data indicate the centrality of CgCmr1 to functioning of the HR machinery, and establish CgCmr1 to be a pivotal but not the sole regulator of the HR pathway and MMS-induced DNA damage response in *C*. *glabrata*. Considering this, we hypothesize that the MMS resistance and highly efficient HR system in the *Cghhf1Δhhf2Δ* mutant could stem from higher abundance of HR factors, and that, CgCmr1 may be required for the recruitment of these HR factors on chromatin to start the repair of MMS-induced DNA damage. However, it must be noted that since low H4 dosage is associated with MMS resistance, the reduced histone H4 levels, increased MMS susceptibility and diminished HR efficiency in the *Cgcmr1Δ* mutant could also reflect two independent roles of CgCmr1 in histone H4 homeostasis and HR pathway.

## Discussion

*C*. *glabrata*, a pathogen of high clinical relevance, shares several physiological features with the non-pathogenic yeast *S*. *cerevisiae* [19,23]. Its ability to proliferate inside host macrophages is in part dependent upon the remodelled chromatin and activated DNA damage response [24]. Here, through genetic, transcriptional and proteomic analyses, we have delineated the mechanisms, by which *C*. *glabrata* responds to the MMS-induced DNA damage, and report four new findings. First, HR pathway is essential for survival of the MMS stress. Second, the WD-repeat protein CgCmr1, a histone H4 interactor, is pivotal to MMS tolerance, proper functioning of the HR machinery and maintenance of H4 levels. Third, the reduced histone H4 dosage leads to a more efficient HR pathway and faster repair of the damaged DNA, thereby, rendering cells less susceptible to MMS. Lastly, *C*. *glabrata* cells respond to MMS by reducing the number of protein interactions for the histone H4 protein. Notably, this is the first report to link low histone H4 dosage with MMS resistance, though H4 depletion [52], and simultaneous depletion of H3 and H4, [53] have previously been shown to result in increased HR, and increased HR and DNA damage resistance respectively, in *S*. *cerevisiae*.

Histones are encoded by multiple genes in eukaryotic cells [8]. Three different ORFs in *C*. *glabrata* code for the identical histone H4 protein. Our data demonstrate for the first time differential effects of these ORFs on the MMS-induced DNA damage response in *C*. *glabrata*, as *CgHHF1* and *CgHHF2* gene loss rendered cells MMS resistant, while *CgHHF2* and *CgHHF3* gene loss led to MMS susceptibility (Fig 1B). To better understand this paradoxical result, we performed Western analysis to determine H4 levels in MMS-treated *Cghhf1Δhhf2Δ* and *Cghhf2Δhhf3* cells and found a 20% difference in total histone H4 levels between these two mutants, with *Cghhf2Δhhf3* cells showing lower H4 levels (S8 Fig). Whether this small difference in H4 amount is sufficient to account for differential MMS susceptibility of *Cghhf1Δhhf2Δ* and *Cghhf2Δhhf3* mutants remains to be determined. We believe that MS-based absolute quantification of total, cytosolic and nuclear histone H4 levels in MMS-treated *wt*, *Cghhf1Δhhf2Δ* and *Cghhf2Δhhf3Δ* cells is warranted to unequivocally correlate H4 levels with differential MMS susceptibility.

The low histone H4 dosage can impact many facets of the DNA metabolism including nucleosome density, replication fork stability, interaction of chromatin-modifying enzymes and activation of the DNA damage response [2,4,21]. Our data suggest a negative regulation of the HR pathway by histone H4, as higher HR efficiency was found to be associated with low H4 dosage in the *Cghhf1Δhhf2Δ* mutant [Fig 3B]. Notably, histones H3 and H4 in *S*. *cerevisiae* have previously been shown to compete with HR factors [53]. Further, the low histone H4 dosage appears to have counterintuitive effects on cellular physiology of *C*. *glabrata*, with increased ribosomal translational capacity and decreased 'de novo' IMP biosynthetic process (S2 Table). This paradoxical outcome could either simply reflect the differential effect of H4 abundance on transcript expression or indirect effects of low H4 stress. Determining whether the effect of histone H4 dosage on the number, position and/or occupancy time of nucleosomes is gene specific or genome-wide may aid in understanding this enigmatical result.

**Fig 7:**
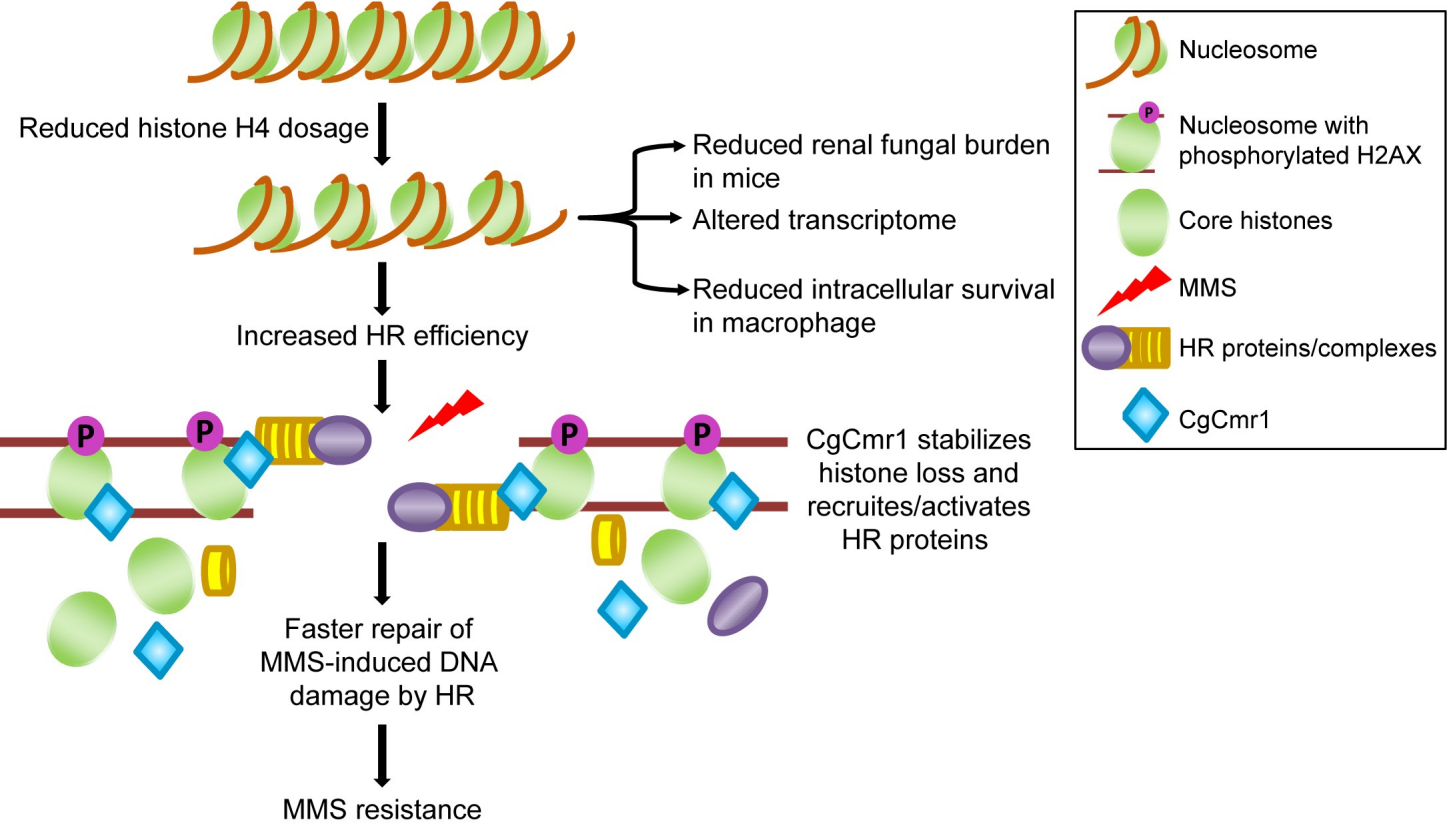
Histone H4 gene dosage is linked with MMS tolerance in *C*. *glabrata*. A schematic depicting the nexus among H4, CgCmr1 and repair of MMS-induced DNA damage. *C*. *glabrata* with low histone H4 dosage showed increased homologous recombination efficiency, altered gene expression, and attenuated survival in macrophages and mice. The low H4 dosage may render the chromatin more accessible for binding of HR proteins resulting in faster repair of the damaged DNA. CgCmr1 stabilizes histone H4 levels, and may aid in the recruitment/activation of HR proteins for DNA repair.

Why do histone levels drop under DNA-damaging conditions? Our data suggest that a reduction in H4 levels upon MMS exposure may be beneficial in three respects: increased chromatin accessibility to the DNA damage repair factors, temporary tilting the balance of competition in favour of HR factors, and increased availability of CgCmr1 to initiate the DNA damage recovery process probably though re-localization of DNA repair and recombination system components. Moreover, since both H3 and H4 associate with CgCmr1 under normal and MMS-treatment conditions, diminished H3 levels in MMS-treated cells may also confer same advantages under MMS stress. Further, although *CgCMR1* transcript levels remained unchanged upon MMS treatment (S7C Fig), CgCmr1 was found to be required for MMS response and HR pathway. Based on these data, we hypothesize that H4-CgCmr1 interaction happens on chromatin, and this association prevents H4 degradation and stabilizes its levels (Fig 7). The reduced histone H4 levels have a positive impact on binding of HR factors to chromatin. Additionally, CgCmr1 regulates the activity and/or recruitment of HR factors to the chromatin, and CgCmr1-H4 association on chromatin may aid in this process (Fig 7). It is notable that Cmr1 in *S*. *cerevisiae* is known to interact with all core histones [54], and mobilize proteins regulating multiple processes including DNA repair to the intranuclear quality control (INQ) compartment and form foci under genotoxic stress [50]. However, unlike CgCmr1, ScCmr1 is not required to survive MMS stress [49] pointing toward some functional diversity between these two proteins.

Do pathogenic fungi respond transcriptionally differentially to the DNA damage response? To address this question, we closely inspected the published data and compared the MMS response of *S*. *cerevisiae* and *C*. *glabrata*. This comparative transcriptome analysis revealed a largely similar response with few peculiar features. The transcriptional response of *S*. *cerevisiae* to MMS included differential expression of DNA repair and recombination genes and protein degradation machinery [55,56]. Of note, we found *LYS20* (involved in lysine biosynthesis and DNA damage repair), *MAG1* (involved in base excision repair pathway) and *RAD26*, *DUN1*, and *RTT107* (involved in DNA repair) genes to be induced in both yeasts upon MMS treatment (S4 Table) [55,56]. Further, like *C*. *glabrata*, transcription of genes encoding ergosterol biosynthesis enzymes and mitochondrial electron transport system components were repressed in MMS-treated *S*. *cerevisiae* cells [56], underscoring a similar transcriptional response of two closely related yeasts to the alkylating agent MMS. However, few key differences were noticed as well. Unlike, *S*. *cerevisiae*, induction of the protein degradation response was subdued in MMS-treated *C*. *glabrata*, with only ubiquitin-specific protease-encoding *CgUBP11* gene showing upregulation (S4 Table). Similarly, the wholesale upregulation of amino acid metabolism genes, and downregulation of rRNA synthesis and ribosomal proteins was not observed in MMS-exposed *C*. *glabrata* cells (S6 Table). Markedly, of 9 specific DNA damage signature genes in *S*. *cerevisiae* [56], ortholog of only one gene, *DUN1*, was found to be induced in *C*. *glabrata* upon MMS exposure (S4 Table). Collectively, these data indicate that despite some commonality, the transcriptional response of *C*. *glabrata* to MMS is distinct.

Finally, does the *in vitro* MMS sensitivity/resistance phenotype govern the *in vivo* outcome? To address this question, we examined the survival of *Cghhf1Δhhf2Δ* (MMS-resistant), *Cgcmr1Δ* (MMS-sensitive), *Cgrad52Δ* (MMS-supersensitive), *Cgyku80Δ* (wild-type-like MMS-sensitive) mutants in human THP-1 macrophages and mice. We found that while CgKu80, CgRad52 and CgCmr1 loss had no effect on the intracellular proliferation in macrophages and survival in mice, *C*. *glabrata* cells with low histone H4 dosage exhibited a 2-fold replication defect in THP-1 macrophages (S9A Fig), and 5-fold lower colony-forming units in kidneys of infected mice (S9B Fig). Besides suggesting that the enhanced MMS stress survival phenotype of the *Cghhf1Δhhf2Δ* mutant was not translated into better fitness *in vivo*, these data also raise the possibility that positive effect of the low H4 dosage on HR efficiency may be repealed by adverse effects on the cellular metabolism in the mice infection model. Collectively, these results uncouple the *in vitro* and *in vivo* consequences of gene loss in *C*. *glabrata*, and underscore the multifaceted complex mechanisms required to survive and establish infections in the mammalian host.

In conclusion, besides establishing CgCmr1 as a pivotal determinant of the HR pathway, our data suggest that the abundance of histone H4 acts as a regulatory cue for the MMS stress response and expression of virulence factors in *C*. *glabrata*.

## Materials and methods

### Ethics statement

Mice infection experiments were conducted at the Animal House Facility of Centre for DNA Fingerprinting and Diagnostics (CDFD; www.cdfd.org.in) and VIMTA Labs Limited (http://www.vimta.com), Hyderabad, India in accordance with guidelines of the Committee for the Purpose of Control and Supervision of Experiments on Animals, Government of India. The protocols were approved by the Institutional Animal Ethics Committee [PCD/CDFD/05—VIMTA Labs and EAF/RK/CDFD/22—CDFD]. Procedures used in these protocols were designed to minimize animal suffering.

### Strains and media

The YPD medium was used to culture *C*. *glabrata wild-type* and mutant strains, which are derivatives of the vaginal isolate BG2. Bacterial strains were grown in the LB medium at 37°C. Logarithmic (log)-phase *C*. *glabrata* cells were obtained after growing overnight cultures in the fresh medium at 30°C for 4 h.

### *C*. *glabrata* gene cloning and disruption

All *C*. *glabrata* deletion strains were generated, using the homologous recombination-based approach, with the *nat1* gene (confers nourseothricin resistance) as a selection marker, as described previously [57]. Replacement of the disrupted ORF with the FRT-*nat1* cassette was confirmed by PCR. To create the double and triple deletion strains, using the same *nat1* gene, the *nat1* gene was first flipped out from the single/double deletion mutant by expressing the gene encoding flip recombinase, which utilizes FRT sites present in the FRT-*nat1* cassette, from a plasmid. The resultant nourseothricin sensitive colonies were used for disruption of another ORF through homologous recombination. The *Cghhf1Δ*, *Cghhf3Δ*, *Cghht1Δ* and *Cghht2Δ* strains were used to generate *Cghhf1Δ2Δ*, *Cghhf2Δ3Δ*, *Cghht1Δ2Δ*, *Cghht2Δ3Δ* double-deletion strains, respectively. For pair-deletion strain creation, the whole H3-H4 gene pair was replaced with the *nat1* gene on the chromosome. The double-paired deletion strain was generated by disrupting the *CgHHT1-HHF1* gene pair in the *Cghht2Δ-hhf2Δ* parental strain background. Lastly, H3- and H4-overexpressing strains were generated via ectopic expression of *CgHHT2* and *CgHHF1* genes, respectively, from the strong *CgPDC1* promoter. Histone mutant complementation studies were performed by expressing *C*. *glabrata* genes ectopically from the *CgPDC1* promoter. For histone H4 ORF expression from their native promoters, *CgHHF1* gene along with the 947 bp upstream sequence, *CgHHF2* gene along with the 952 bp upstream sequence and *CgHHF3* gene along with the 988 bp upstream sequence were amplified from genomic DNA of *Cghhf2Δ3Δ*, *wild-type* and *wild-type* strains, respectively. The amplified *CgHHF1*, *CgHHF2* and *CgHHF3* fragments were individually cloned in SacI and XmaI restriction enzyme sites in the pGRB2.1 plasmid.

For epitope tagging of *C*. *glabrata* proteins at the C-termini, the *CgHHF3* (*CAGL0H09834g*; 0.31 kb), *CgHHT1* (*CAGL0C04114g*; 0.41 kb), *CgCMR1* (*CAGL0I03542g*; 1.60 kb), *CgSPT16* (*CAGL0C03047g*; 3.01 kb) and *CgPSH1* (*CAGL0I09988g*; 1.12 kb) genes were cloned in SpeI-XmaI, XbaI-SpeI, SpeI-XmaI, XbaI-XmaI and SpeI-XmaI sites in the pRK1349 plasmid, respectively, between the *CgPDC1* promoter and the triple epiotpe SFB (S protein-Flag-Streptavidin-binding peptide)-encoding sequence. The *CgRAD52* (*CAGL0G07381g*; 1.52 kb) gene was cloned in SpeI-XmaI sites in the pRK1018 plasmid between *PGK1* promoter and GFP-encoding sequence, and the resultant plasmids were transformed into *C*. *glabrata* strains. Strains, plasmids and primers used in this study are listed in S13, S14 and S15 Tables, respectively.

### Protein extraction and immunoblotting

Log-phase-grown *C*. *glabrata* cells were grown in the medium lacking or containing MMS for different time intervals and harvested. Cells were lysed with glass beads in the protein extraction buffer [50 mM Tris-HCl (pH 7.5), 2 mM EDTA, 10 mM sodium fluoride, 1 mM sodium orthovanadate and 1 X protease inhibitor] and spun down at 13000 rpm for 15 min at 4°C. 50 μg protein were run on a 15% SDS-PAGE gel and immunoblotted with appropriate antibodies. For affinity purification, log-phase *C*. *glabrata* cells expressing either the SFB epitope or SFB-tagged yeast proteins ectopically were grown in the CAA medium lacking or containing 0.06% MMS for 3 h and collected. 3.5 mg protein equivalent cell lysates, prepared by glass bead-beating, were incubated with streptavidin beads for 2 h at 4°C. After washing, beads were boiled in 2X SDS dye, proteins resolved on a 15% SDS-PAGE gel, and immunoblotted with appropriate antibodies. Antibodies used in this study are listed in S16 Table.

### DNA damage recovery assay

Log-phase cultures of *wild-type* (*wt*) and *Cghhf1Δ2Δ* strains were treated with 0.06% MMS for 45 min and cells were harvested. After two washes, cells were suspended in the fresh YPD medium and incubated at 30°C for recovery from MMS stress. Samples were collected at every 90 min interval till 9 h, whole cell extracts prepared and γ-H2AX levels were detected using the anti-γH2AX antibody.

### RNA-sequencing analysis

YPD-grown log-phase cultures of *wt* and *Cghhf1Δ2Δ* strains were either treated with 0.06% MMS for 45 min or left untreated. Cells were collected and total RNA was extracted using the acid phenol method. After DNaseI treatment to remove DNA contamination, frozen RNA samples were sent to the Scigenom, Kochi, India (http://www.scigenom.com/) for library preparation and sequencing. Libraries were prepared using the Truseq RNA library prep kit, and 100 bp paired-end sequencing was performed on Illumina Hiseq 2500. Sequence reads were processed, aligned to the *C*. *glabrata* CBS138 reference genome and quantified using the reference gene model in Kallisto v0.44.0. The DESeq program was used to identify differentially expressed genes, that showed ≥ 2-fold change in expression and a *p* value of ≤ 0.05.

### Plasmid circularization assay

Log-phase-grown *wt* and *Cghhf1Δ2Δ* cells, which lack the *CgURA3* gene, were transformed either with the circular plasmid pRK74 (100 ng) or the NcoI-linearized pRK74 plasmid (250 ng). Transformants were incubated at 30°C for 48 h and selected for uracil prototrophy. Since the NcoI enzyme cuts in the *CgURA3* gene in the pRK74 plasmid, the number of colonies obtained on the CAA medium lacking uracil reflected the frequency at which cells could circularize the linear plasmid through NHEJ (Non-homologous end joining). The number of uracil prototroph transformants obtained with the circular plasmid indicated the efficiency of DNA uptake. The percentage efficiency of plasmid circularization was calculated by dividing the number of transformants obtained with the linear plasmid by the number of transformants obtained with the circular plasmid, and multiplying this number by 100. A total of 200 transformants were analysed for this assay.

### Homologous recombination efficiency measurement

The frequency of homologous recombination was measured by two assays. In the first assay, log-phase-grown *wt* and *Cghhf1Δ2Δ* strains, wherein the *CgURA3* gene was replaced with the geneticin resistance-conferring gene *neo*, were transformed with the PCR amplified *CgURA3* gene (250 ng) carrying 850 and 700 bp of 5’ and 3’ UTRs (untranslated regions), respectively. Transformants were selected for uracil prototrophy after 4 h growth in the YPD medium, allowing homologous recombination at the *CgURA3*-UTRs to replace the *neo* gene with the *CgURA3* gene in the genome. The total number of uracil prototroph transformants reflected the number of *CgURA3* integration events occurring in the genome. The number of uracil prototroph and geneticin (G418) resistant transformants represented the integration events, that occurred randomly at loci other than *CgURA3*. Homologous recombination frequency was calculated based on the ratio of the total number of uracil prototroph and G418 sensitive colonies to the total number of uracil prototroph transformants. A total of 200 transformants were analysed for this assay.

In the second HR frequency measurement assay, FOA (5-Fluoroorotic acid) resistance was used as a direct read-out of the HR efficiency. In brief, *wt*, *Cghhf1Δhhf2Δ*, *Cgcmr1Δ* and *Cghhf1Δhhf2Δcmr1Δ* strains (uracil prototophs) were transformed with a PCR amplified linear fragment carrying the G418 resistance-conferring gene, *neo*, flanked by 750 bp of *5’UTR* on one end and 550 bp of *3’UTR* of the *CgURA3* gene on the other side, and plated on medium containing FOA. Replacement of the *CgURA3* gene at the genomic locus with the *neo* gene, via homologous recombination at *CgURA3-*UTRs, rendered strains uracil auxotrophs and, thus, FOA resistant. The number of FOA resistant colonies obtained for each strain was counted. For the transformation efficiency control, all strains were transformed with a circular plasmid expressing *hph1* gene and the number of hygromycin resistant transformants was enumerated. HR efficiency was plotted by dividing the total number of FOA resistant colonies by the total number of hygromycin-resistant transformants, and multiplying this number by 100. A minimum of 200 transformants were analysed for this assay.

### Biofilm formation assay

*C*. *glabrata* cells were grown in YPD medium to log-phase and collected. 0.5 OD_600_ cells were seeded in a 24 well polystyrene plate and incubated at 37°C for 90 min. After two PBS washes, RPMI 1640 medium containing 10% FBS was added to each well, and the plate was incubated at 37°C. After 24 h, the spent medium was replaced with the fresh RPMI medium, and the plate was incubated at 37°C for another 24 h. After removing unbound *C*. *glabrata* cells with three PBS washes, crystal violet [0.4% (w/v)] was used to stain the adherent cells. The 45 min crystal violet staining was followed by destaining with 95% ethanol. The absorbance of the destaining solution was measured at 595 nm, and absorbance values of blank wells (without *C*. *glabrata*) were subtracted from those of *C*. *glabrata*-containing wells. Data were plotted as biofilm ratio, which represents the mutant/*wt* absorbance units.

### Two-step affinity purification and mass spectrometry analysis

Log-phase cells of *Cghht1Δ2Δ/CgHHT1-SFB* and *Cghhf1Δ2Δ-CgHHF3-SFB* strains were grown for 3 h in the CAA medium lacking or containing 0.06% MMS. Both strains retained 100% viability during this treatment period. Cells were harvested, lysed with glass beads, and cell lysates (5 mg) were incubated with streptavidin beads for 2 h at 4°C with constant rocking. Following centrifugation and washes, beads were incubated with the biotin solution (2 mg/ml) for 2 h at 4°C. Next, beads were spun down, and the supernatant was incubated with S-protein agarose beads for 2 h at 4°C. Post centrifugation and washes, beads were boiled for 5 min in 2X SDS dye and run on a 10% SDS-PAGE gel. Gel was stained with Coomassie Brilliant Blue, and protein bands were cut and sent from two biological replicate samples to the Taplin Biological Mass Spectrometry Facility, Harvard Medical School (https://taplin.med.harvard.edu) for LC-MS/MS analysis. Peptides were identified using the Sequest software, filtered to 1% false discovery rate and mapped to the *C*. *glabrata* reference proteome database (www.candidagenome.org/). Proteins identified with ≥ 2 total peptides in both biological replicate samples were chosen for further analysis. For isolation of specific interactors of histones H3 and H4, proteins, that bound non-specifically to the affinity matrices, and were identified in the SFB-epitope expressing *Cghht1Δ2Δ* and *Cghhf1Δ2Δ* strains, were removed from the H3 and H4 interactor list.

### Other procedures

Stress susceptibility, quantitative Real-time PCR (qPCR), and macrophage and mice infection analysis were performed, as described previously [24,57].

### Statistical and functional analysis

The GraphPad Prism software was used to check the statistical significance of differences observed using the two-tailed Student’s t-test for intergroup comparisons. *C*. *glabrata* genes were functionally annotated to different processes using the Candida Genome Database (CGD)-GO (gene ontology) Slim Mapper tool for BP (biological process) (http://www.candidagenome.org/cgi-bin/GO/goTermMapper). The GO functional enrichment analysis was carried out using the FungiFun tool (https://elbe.hki-jena.de/fungifun/), with *C*. *glabrata* CBS138 as the reference strain.

### Data availability

The raw RNA-Seq data are deposited in the NCBI's Gene Expression Omnibus [58] with the GEO accession number GSE142737. The raw mass spectrometry data are deposited in the ProteomeXchange Consortium via the PRIDE [59] partner repository with the dataset identifier PXD016867.

## Supporting information

S1 Table List of differentially expressed genes in the *Cghhf1Δhhf2Δ* mutant. (XLSX)

S2 Table GO-BP enrichment analysis of differentially expressed genes in the *Cghhf1Δhhf2Δ* mutant. (XLSX)

S3 Table List of differentially expressed subtelomeric genes in the *Cghhf1Δhhf2Δ* mutant. (XLSX)

S4 Table List of differentially expressed genes in MMS-treated *wild-type* cells. (XLSX)

S5 Table List of differentially expressed genes in MMS-treated *Cghhf1Δhhf2Δ* cells. (XLSX)

S6 Table GO-BP enrichment analysis of differentially expressed genes in MMS-treated *wild-type* cells. (XLSX)

S7 Table GO-BP enrichment analysis of differentially expressed genes in MMS-treated *Cghhf1Δhhf2Δ* cells. (XLSX)

S8 Table List of histone H4 interacting proteins. (XLSX)

S9 Table List of histone H3 interacting proteins. (XLSX)

S10 Table GO-BP enrichment analysis of the common interactors of histones H3 and H4. (XLSX)

S11 Table GO-BP enrichment analysis of the histone H4 interactors. (XLSX)

S12 Table GO-BP enrichment analysis of the histone H3 interactors. (XLSX)

S13 Table List of *C. glabrata* strains used in the study. (XLSX)

S14 Table List of plasmids used in the study. (XLSX)

S15 Table List of primers used in the study. (XLSX)

S16 Table List of antibodies used in the study. (XLSX)

S17 Table Raw numerical data underlying plotted graphs. (XLSX)

S1 Fig The histone H3 and H4 are encoded by three different ORFs in *C. glabrata*. **A.** Schematic representation of histone H3- and H4-encoding gene loci on chromosomes of *C*. *glabrata* and *S*. *cerevisiae*. The synteny between *C*. *glabrata* and *S*. *cerevisiae* histone H3- and H4-encoding ORFs was determined using the YGOB tool (http://ygob.ucd.ie). Histone H3- and H4-encoding ORFs are highlighted in cyan coloured boxes. **B.** Amino acid sequence alignment of the histone H3 protein encoded by *CAGL0C04114g*, *CAGL0H09856g* and *CAGL0M06655g* ORFs in *C*. *glabrata*. The Clustal Omega multiple sequence alignment tool (https://www.ebi.ac.uk/Tools/msa/clustalo/) was used for this analysis. Black asterisk indicates identical amino acid residue. **C.** Amino acid sequence alignment of the histone H4 protein encoded by *CAGL0C04136g*, *CAGL0H09834g* and *CAGL0M06677g* ORFs in *C*. *glabrata*. The Clustal Omega multiple sequence alignment tool (https://www.ebi.ac.uk/Tools/msa/clustalo/) was used for this analysis. Black asterisk indicates identical amino acid residue. **D.** A list of the number of histone H3- and H4-encoding ORFs in eleven fungal species. Histone H3 and H4 ORFs in species of the *Nakaseomyces* genus were identified through BLASTP, using *S*. *cerevisiae* H3 and H4 protein sequences as query, against the Genome Resources for Yeast Chromosome (GRYC) database (http://gryc.inra.fr). For *Aspergillus nidulans*, *C*. *albicans*, *C*. *tropicalis*, *C*. *auris*, *Cryptococcus neoformans* and *Histoplasma capsulatum*, BLASTP was run against the FungiDB database (https://fungidb.org/fungidb), using *S*. *cerevisiae* H3 and H4 protein sequences as query.
(TIF)

S2 Fig The *Cghhf1Δhhf2Δ* mutant displays resistance to MMS. **A.** Time course analysis. Indicated strains were grown overnight in the YPD medium, and re-inoculated in the fresh YPD medium at an initial OD_600_ of 0.1. Cultures were incubated at 30⁰C with shaking (200 rpm) in a shaker-incubator. Absorbance of each culture was recorded at regular intervals till 36 h, and plotted against the time. Data represent mean ± SEM of 3-independent experiments. The doubling time was calculated during the log-phase (2–8 h of growth period) of cultures. Differences in the doubling time of *wt* and *Cghht1Δhht2Δ*, *wt* and *Cghht2Δhht3Δ*, *wt* and *Cghhf2Δhhf3Δ*, and *wt* and *Cghht-hhf1Δ2Δ* strains, were found to be statistically significant. *, p ≤ 0.05; unpaired two-tailed Student’s t test. **B.** Serial dilution spot assay showing thermal stress sensitivity and MMS resistance of the *Cghhf1Δhhf2Δ* mutant to be rescued upon ectopic expression of each one of the three *CgHHF* genes from their respective native promoters. Growth of *C*. *glabrata* cultures was recorded after 1 day incubation at 42°C. For YPD and MMS, plates were incubated at 30°C and photographed after day 2 for YPD, 0.04% and 0.05% MMS, and day 3 for 0.06% MMS. **C.** Serial dilution spot assay showing MMS resistance of four independently generated *Cghhf1Δhhf2Δ* mutants. This resistance was brought down to *wt* level upon ectopic expression of the *CgHHF1* gene. Growth of *C*. *glabrata* cultures was recorded after 1 day incubation at 42°C. For YPD and MMS, plates were incubated at 30°C and photographed after day 2 for YPD, 0.04% and 0.05% MMS, and day 3 for 0.06% MMS.
(TIF)

S3 Fig The *Cghhf1Δ2Δ* mutant is not resistant to oxidative stress. **A.** Serial dilution spot assay displaying growth of indicated strains in the presence of genotoxic and oxidative stressors. The thymine dimerization-causing ultraviolet radiation (UV; 50 and 100 J/m^2^), and ribonucleotide reductase inhibitor hydroxyurea (HU; 200 and 500 mM) were used as genotoxic stressors. The hydrogen peroxide (H_2_O_2_; 25 and 45 mM) was used as an oxidative stress-causing agent. Images were captured after 2 days’ incubation at 30°C. **B.** Serial dilution spot assay showing that histone H4 (*CgHHF1*) and H3 (*CgHHT2*) overexpression did not alter MMS and thermal stress susceptibility of *wt* cells. *wt*/V refers to the *wt* strain carrying the empty vector. **C.** Serial dilution spot assay showing increased and decreased susceptibility of the *Cghht1-hhf1Δhht2-hhf2Δ* mutant, that lacks two pairs of canonical H3-H4 genes, to thermal stress and MMS stress, respectively, compared to *wt* cells. **D.** qPCR-based measurement of histone H3 (*CgHHT*) and H4 (*CgHHF*) transcript levels in indicated histone H3- and H4-deleted mutants. *C*. *glabrata* strains were either left untreated or treated with 0.06% MMS for 45 min. Data (mean ± SEM, *n =* 3) were normalized against the *CgACT1* mRNA control, and represent fold change in *CgHHT* and *CgHHF* expression in treated samples compared to untreated samples (taken as 1.0). *, *p* ≤ 0.05, **, *p* ≤ 0.01; paired two-tailed Student’s *t* test. **E.** Colony forming unit (CFU)-based viability analysis. *wt* and *Cghhf1Δ2Δ* strains were grown in the YPD medium for 3 h and treated with 0.06% MMS. At indicated time points, cells were collected and appropriate dilutions were plated on the YPD medium. After 2 days’ incubation at 30°C, CFUs were counted. The percentage survival for each strain was calculated by dividing the number of CFUs at indicated time point by the number of CFUs prior to MMS addition (0 h), and multiplying this number by 100. Data (mean ± SEM, *n =* 3) are plotted as a line graph. *, *p* ≤ 0.05, **, *p* ≤ 0.01; unpaired two-tailed Student’s *t* test.
(TIF)

S4 Fig Schematic representation depicting the domain and nuclear localization sequence (NLS) present in the *C. glabrata* and *S. cerevisiae* Rad52 protein. The Rad52_Rad22 domain was identified at the N-termini of both proteins using the Pfam tool (http://pfam.xfam.org). The NLS mapper tool (http://nls-mapper.iab.keio.ac.jp) predicted one bipartite NLS at the C-terminus of ScRad52, and two NLSs in the CgRad52 protein, with one monopartite NLS at the middle and one bipartite NLS at the C-terminus. The cut-off score was set to 3.0 for NLS prediction analysis.
(TIF)

S5 Fig The *Cghhf1Δ2Δ* mutant displayed increased susceptibility to micrococcal nuclease (MNase) digestion. An equal number of log-phase *wt* and *Cghhf1Δ2Δ* cells were collected, suspended in spheroplasting buffer [50 mM Tris Cl (pH 7.5) and 1 M sorbitol] and treated with zymolyase (1 mg/10 OD_600_ cells) for 30 min at 37°C. The generated spheroplasts were suspended in MNase-digestion buffer [10 mM Tris (pH 8.0) and 1 mM CaCl_2_] and digested with 10 units of MNase at 37°C. Digested samples were harvested at indicated time points, and DNA was isolated using the phenol-chloroform extraction method. 12 μg of purified DNA were resolved on 1.4% agarose gel and stained using ethidium bromide. Marker M1 and M2 indicate 100 bp (NEB #N3231L) and 1 kb (NEB #3232L) DNA ladder, respectively.
(TIF)

S6 Fig The SFB-tagged histone H3 and H4 proteins are functional. **A.** An immunoblot showing expression of histone H3 (CgHht1) and H4 (CgHhf3) proteins tagged with the triple SFB epitope at their C-termini. The *Cghht1Δ2Δ* and *Cghhf1Δ2Δ* mutants were transformed with plasmids expressing *CgHHT1-SFB* and *CgHHF3-SFB*, respectively. Transformants were grown in the CAA medium till log-phase, and whole-cell extracts were prepared by glass bead lysis. 50 μg protein were resolved on 15% SDS-PAGE and probed with anti-FLAG antibody. The bands of 27 kDa and 32 kDa correspond to H4-SFB and H3-SFB histone proteins, respectively. **B.** Serial dilution spot assay showing that CgHht1-SFB and CgHhf3-SFB could restore the thermal stress sensitivity, and thermal stress sensitivity and MMS resistance of *Cghht1Δ2Δ* and *Cghhf1Δ2Δ* mutants, respectively. **C.** Bar graphs displaying overlap between the interactomes of *C*. *glabrata* and *S*. *cerevisiae* histone H3 and H4 proteins. The *S*. *cerevisiae* interactome information was obtained from the BioGRID interaction database (https://thebiogrid.org/).
(TIF)

S7 Fig The *Cgcmr1Δ* mutant displays transcriptional downregulation of the histone H4 gene upon MMS treatment. **A.** Schematic illustration of the domain organization of *C*. *glabrata* and *S*. *cerevisiae* Cmr1 protein. Information for this analysis was obtained from the Uniprot Database (https://www.uniprot.org). ScCmr1 has seven WD40 repeats, while CgCmr1 protein contains six WD40 repeats. **B.** qPCR-based measurement of histone H4 transcripts in the untreated and MMS-treated *Cgcmr1Δ* mutant. YPD medium-grown, log-phase cultures of *wt* and *Cgcmr1Δ* strains were either left untreated or treated with 0.06% MMS for 45 min, and RNA was extracted using the acid phenol method. Transcript levels of the *CgHHF* gene were measured by qPCR. Data (mean ± SEM, *n =* 3) were normalized against the *CgACT1* mRNA control, and represent fold change in H4 gene expression in the *Cgcmr1Δ* mutant (*Cgcmr1Δ*_*UT*) compared to *wt* cells (considered as 1.0), and in MMS-treated *Cgcmr1Δ* cells (*Cgcmr1Δ*_*T*) compared to untreated *Cgcmr1Δ* cells (considered as 1.0). **, *p* ≤ 0.01; paired two-tailed Student’s *t* test. Please note that *CgCMR1* loss had no effect on transcription of the H4 gene. **C.** qPCR-based measurement of *CgCMR1* transcripts in MMS-treated *wt* cells. Log-phase *wt* cultures were grown either in the presence or absence of 0.06% MMS for 45 min, and RNA was extracted using the acid phenol method. *CgCMR1* transcript levels were measured by qPCR. Data (mean ± SEM, *n =* 3) were normalized against the *CgACT1* mRNA control, and represent fold change in *CgCMR1* expression in MMS-treated compared to untreated *wt* sample. Please note that MMS exposure had no effect on transcription of the *CgCMR1* gene.
(TIF)

S8 Fig The *Cghhf2Δ3Δ* mutant showed higher reduction in H4 levels upon MMS treatment. Representative immunoblot showing histone H4 levels in MMS-treated *wt*, *Cghhf1Δ2Δ* and *Cghhf2Δ3Δ* cells. Log-phase cultures were either left untreated or treated with 0.06% MMS for 45 min, whole cell lysates (50 μg) resolved on 15% SDS-PAGE and were probed with anti-H4 and anti-GAPDH antibodies. Data (mean ± SEM, *n =* 3) represent % change in H4 levels compared to *wt* untreated samples (considered as 100), and are plotted as a bar graph on the right side of the blot image. *, *p* ≤ 0.05, **, *p* ≤ 0.01; paired two-tailed Student’s *t* test. *, *p* ≤ 0.05, **, *p* ≤ 0.01; unpaired two-tailed Student’s *t* test (Red asterisks).
(TIF)

S9 Fig The low histone H4 dosage is associated with attenuated virulence in the murine model of systemic candidiasis. **A.** Intracellular proliferation of indicated *C*. *glabrata* strains in human THP-1 macrophages, as measured by the CFU-based assay. The human monocytic THP-1 cells were treated with phorbol 12-myristate 13-acetate (PMA; 16 nM) for 12 h followed by recovery in the fresh RPMI medium for 12–14 h. YPD-grown overnight cultures of *C*. *glabrata* strains were infected to PMA-differentiated THP-1 macrophages at a MoI (multiplicity) of 1:10. After 2 h incubation, the non-internalized *C*. *glabrata* cells were washed off with PBS, and infection was continued for another 22 h. Infected macrophages were lysed in water at 2 and 24 h post infection, and appropriate dilutions of macrophage lysates were plated on the YPD medium. The number of colonies, that appeared after 1–2 day incubation at 30°C, was counted. Fold replication for each strain was calculated by dividing the number of intracellular *C*. *glabrata* cells recovered at 24 h by that recovered at 2 h. Data represent mean ± SEM (n = 3). ****, p<0.001; unpaired two-tailed Student's t-test. **B.** Survival analysis of indicated *C*. *glabrata* strains in the murine model of systemic candidiasis. *C*. *glabrata* cells were grown overnight in the YPD medium, collected, washed and were suspended in PBS. 100 μl cell suspension (4X10^7^ cells) was injected into the tail vein of six to eight-week-old female BALB/c mice. Mice were sacrificed 7^th^ day post infection and kidneys, liver and spleen were collected. After organ homogenization in PBS, appropriate homogenate dilutions were plated on the YPD medium containing penicillin and streptomycin, and fungal load in mouse organs was determined. CFUs recovered from organs of the individual mouse are represented by diamonds, while bars indicate the CFU geometric mean (n = 6–8) for each organ. **, p<0.01; Mann-Whitney test.
(TIF)
