# Supplementary figures and images for: Histone H4 dosage modulates DNA damage response in the pathogenic yeast Candida glabrata via homologous recombination pathway
Source: PLoS Genet. 2020 Mar 5;16(3):e1008620. doi: 10.1371/journal.pgen.1008620 (PMC7058290; doi:10.1371/journal.pgen.1008620)

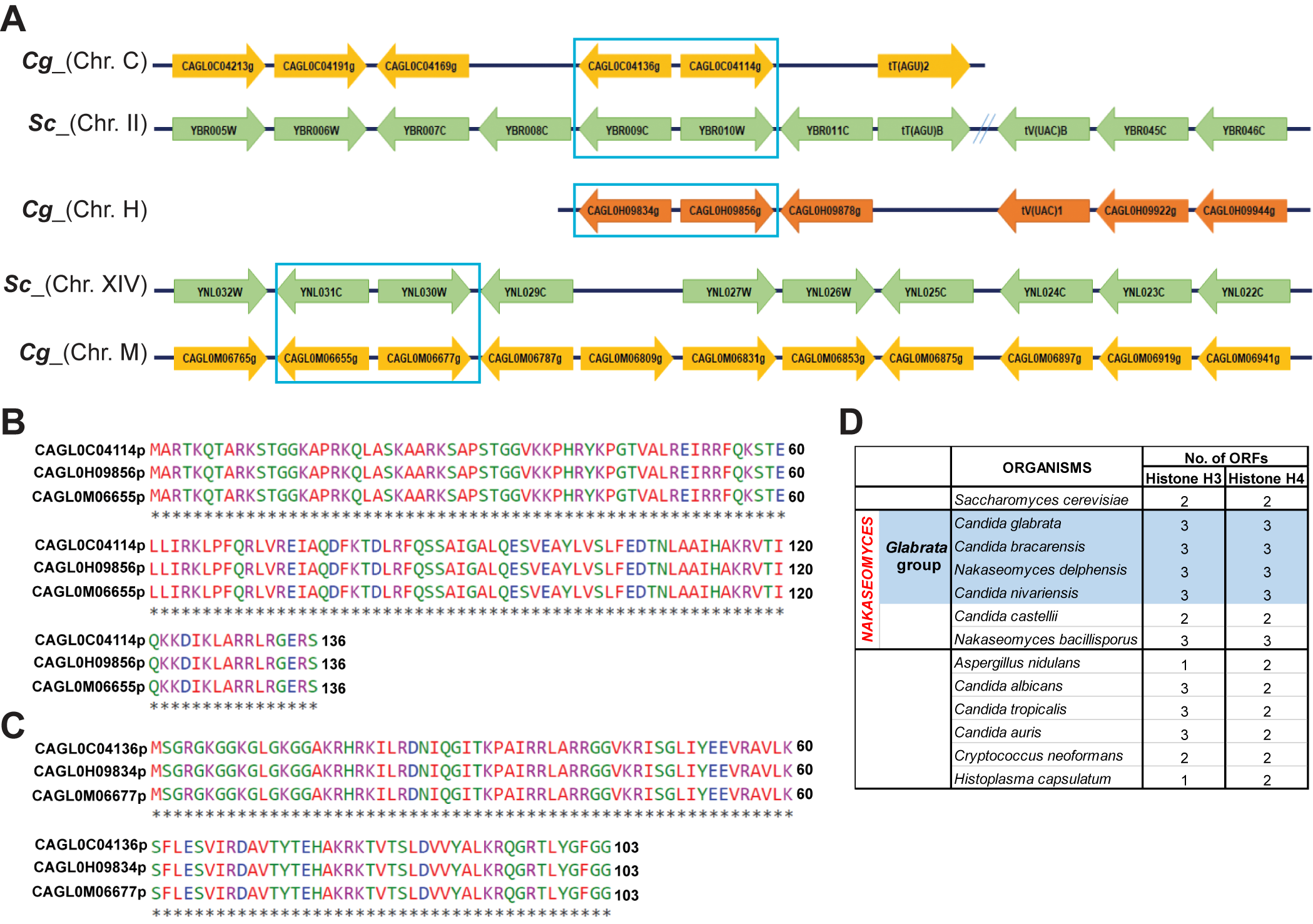

Supplement: S1 Fig — A. Schematic representation of histone H3- and H4-encoding gene loci on chromosomes of C. glabrata and S. cerevisiae. The synteny between C. glabrata and S. cerevisiae histone H3- and H4-encoding ORFs was determined using the YGOB tool (http://ygob.ucd.ie). Histone H3- and H4-encoding ORFs are highlighted in cyan coloured boxes. B. Amino acid sequence alignment of the histone H3 protein encoded by CAGL0C04114g, CAGL0H09856g and CAGL0M06655g ORFs in C. glabrata. The Clustal Omega multiple sequence alignment tool (https://www.ebi.ac.uk/Tools/msa/clustalo/) was used for this analysis. Black asterisk indicates identical amino acid residue. C. Amino acid sequence alignment of the histone H4 protein encoded by CAGL0C04136g, CAGL0H09834g and CAGL0M06677g ORFs in C. glabrata. The Clustal Omega multiple sequence alignment tool (https://www.ebi.ac.uk/Tools/msa/clustalo/) was used for this analysis. Black asterisk indicates identical amino acid residue. D. A list of the number of histone H3- and H4-encoding ORFs in eleven fungal species. Histone H3 and H4 ORFs in species of the Nakaseomyces genus were identified through BLASTP, using S. cerevisiae H3 and H4 protein sequences as query, against the Genome Resources for Yeast Chromosome (GRYC) database (http://gryc.inra.fr). For Aspergillus nidulans, C. albicans, C. tropicalis, C. auris, Cryptococcus neoformans and Histoplasma capsulatum, BLASTP was run against the FungiDB database (https://fungidb.org/fungidb), using S. cerevisiae H3 and H4 protein sequences as query. (TIF) [file pgen.1008620.s018.tif]

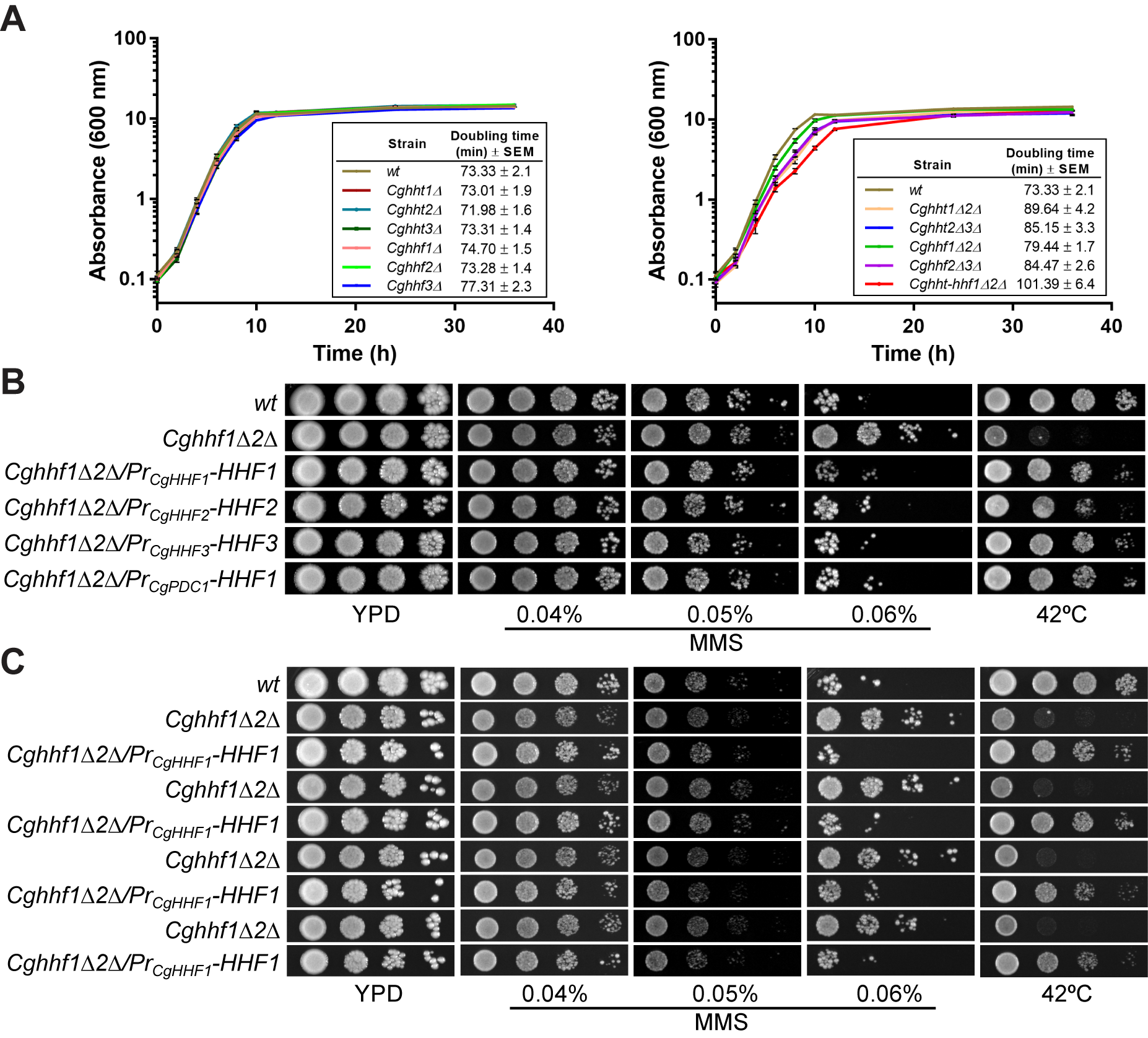

Supplement: S2 Fig — A. Time course analysis. Indicated strains were grown overnight in the YPD medium, and re-inoculated in the fresh YPD medium at an initial OD600 of 0.1. Cultures were incubated at 30⁰C with shaking (200 rpm) in a shaker-incubator. Absorbance of each culture was recorded at regular intervals till 36 h, and plotted against the time. Data represent mean ± SEM of 3-independent experiments. The doubling time was calculated during the log-phase (2–8 h of growth period) of cultures. Differences in the doubling time of wt and Cghht1Δhht2Δ, wt and Cghht2Δhht3Δ, wt and Cghhf2Δhhf3Δ, and wt and Cghht-hhf1Δ2Δ strains, were found to be statistically significant. *, p ≤ 0.05; unpaired two-tailed Student’s t test. B. Serial dilution spot assay showing thermal stress sensitivity and MMS resistance of the Cghhf1Δhhf2Δ mutant to be rescued upon ectopic expression of each one of the three CgHHF genes from their respective native promoters. Growth of C. glabrata cultures was recorded after 1 day incubation at 42°C. For YPD and MMS, plates were incubated at 30°C and photographed after day 2 for YPD, 0.04% and 0.05% MMS, and day 3 for 0.06% MMS. C. Serial dilution spot assay showing MMS resistance of four independently generated Cghhf1Δhhf2Δ mutants. This resistance was brought down to wt level upon ectopic expression of the CgHHF1 gene. Growth of C. glabrata cultures was recorded after 1 day incubation at 42°C. For YPD and MMS, plates were incubated at 30°C and photographed after day 2 for YPD, 0.04% and 0.05% MMS, and day 3 for 0.06% MMS. (TIF) [file pgen.1008620.s019.tif]

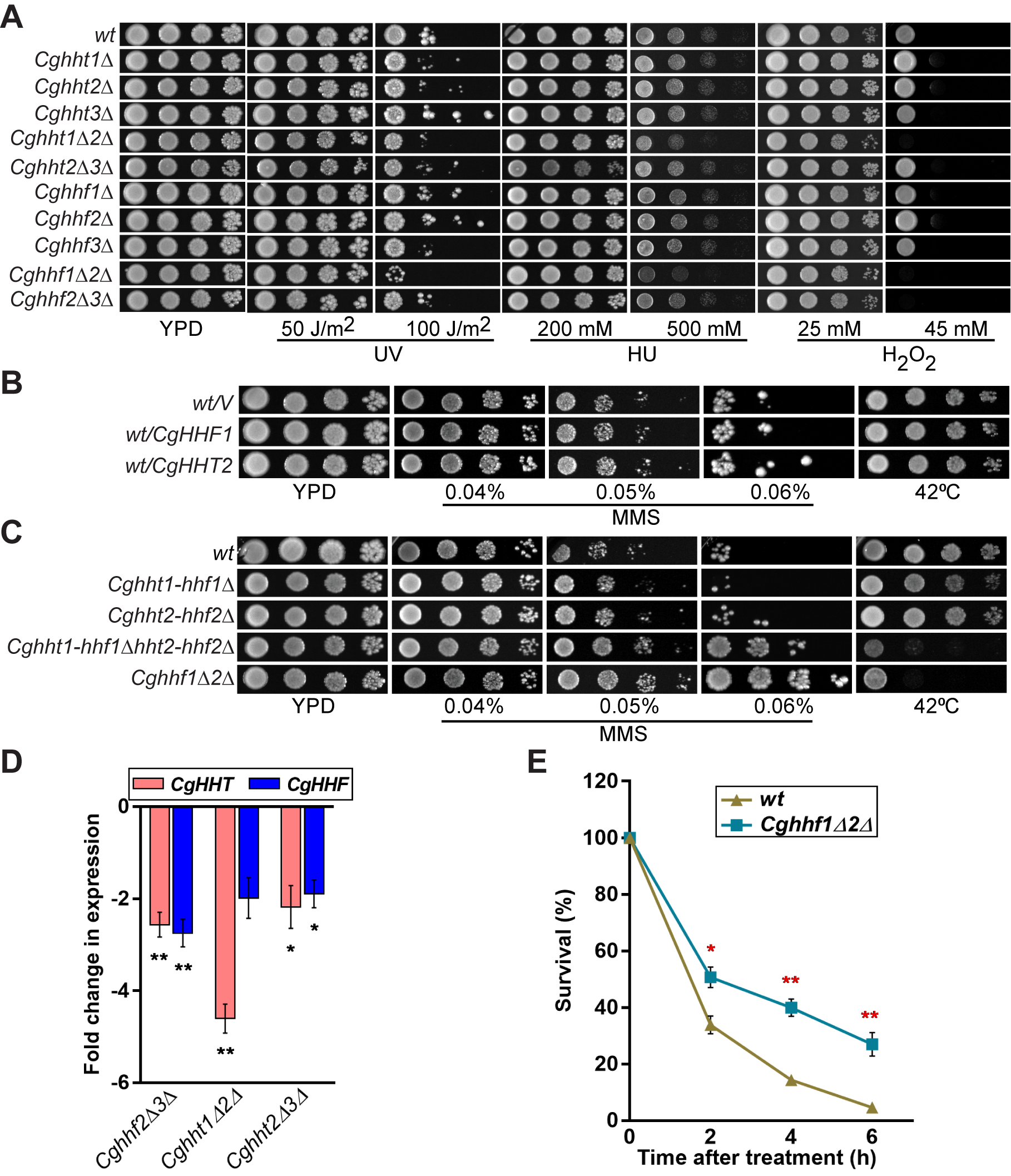

Supplement: S3 Fig — A. Serial dilution spot assay displaying growth of indicated strains in the presence of genotoxic and oxidative stressors. The thymine dimerization-causing ultraviolet radiation (UV; 50 and 100 J/m2), and ribonucleotide reductase inhibitor hydroxyurea (HU; 200 and 500 mM) were used as genotoxic stressors. The hydrogen peroxide (H2O2; 25 and 45 mM) was used as an oxidative stress-causing agent. Images were captured after 2 days’ incubation at 30°C. B. Serial dilution spot assay showing that histone H4 (CgHHF1) and H3 (CgHHT2) overexpression did not alter MMS and thermal stress susceptibility of wt cells. wt/V refers to the wt strain carrying the empty vector. C. Serial dilution spot assay showing increased and decreased susceptibility of the Cghht1-hhf1Δhht2-hhf2Δ mutant, that lacks two pairs of canonical H3-H4 genes, to thermal stress and MMS stress, respectively, compared to wt cells. D. qPCR-based measurement of histone H3 (CgHHT) and H4 (CgHHF) transcript levels in indicated histone H3- and H4-deleted mutants. C. glabrata strains were either left untreated or treated with 0.06% MMS for 45 min. Data (mean ± SEM, n = 3) were normalized against the CgACT1 mRNA control, and represent fold change in CgHHT and CgHHF expression in treated samples compared to untreated samples (taken as 1.0). *, p ≤ 0.05, **, p ≤ 0.01; paired two-tailed Student’s t test. E. Colony forming unit (CFU)-based viability analysis. wt and Cghhf1Δ2Δ strains were grown in the YPD medium for 3 h and treated with 0.06% MMS. At indicated time points, cells were collected and appropriate dilutions were plated on the YPD medium. After 2 days’ incubation at 30°C, CFUs were counted. The percentage survival for each strain was calculated by dividing the number of CFUs at indicated time point by the number of CFUs prior to MMS addition (0 h), and multiplying this number by 100. Data (mean ± SEM, n = 3) are plotted as a line graph. *, p ≤ 0.05, **, p ≤ 0.01; unpaired two-tailed Student’s t test. (TIF) [file pgen.1008620.s020.tif]

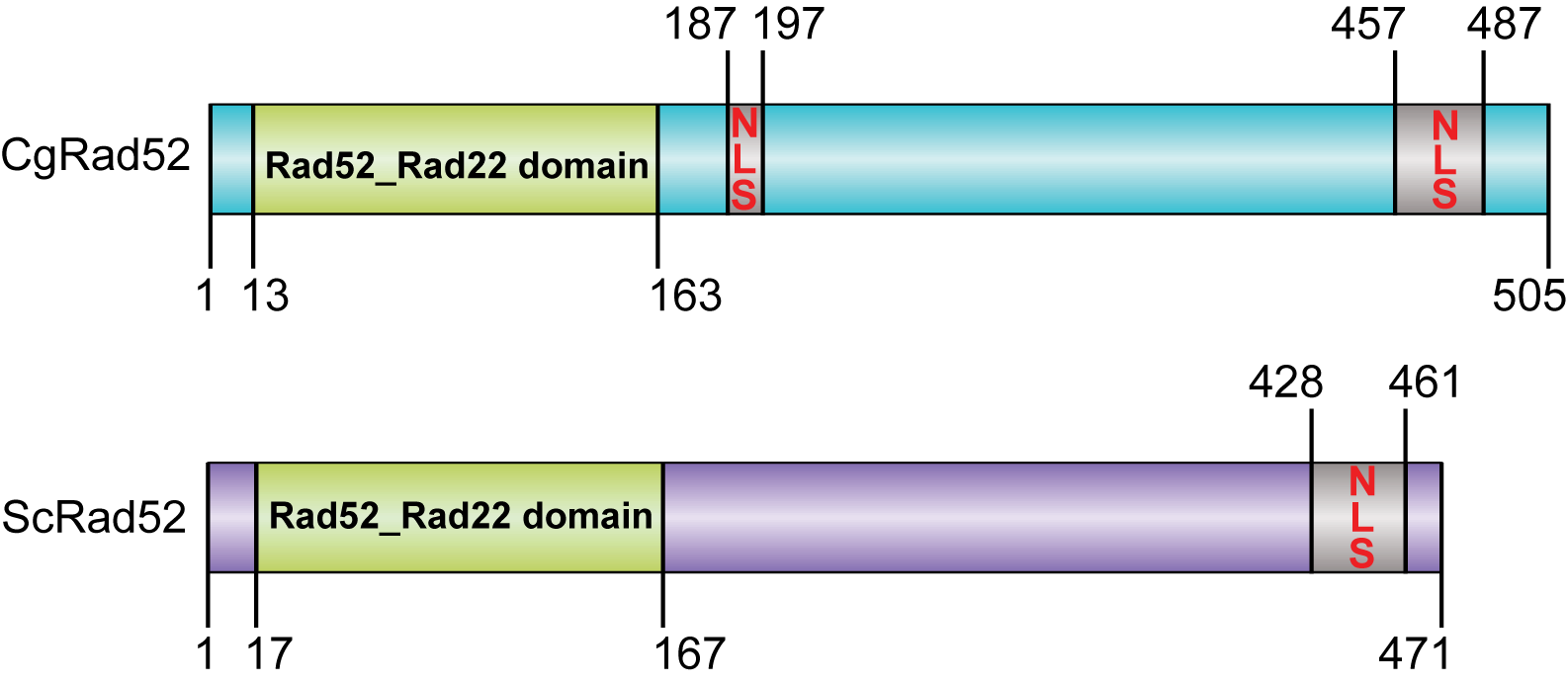

Supplement: S4 Fig — The Rad52_Rad22 domain was identified at the N-termini of both proteins using the Pfam tool (http://pfam.xfam.org). The NLS mapper tool (http://nls-mapper.iab.keio.ac.jp) predicted one bipartite NLS at the C-terminus of ScRad52, and two NLSs in the CgRad52 protein, with one monopartite NLS at the middle and one bipartite NLS at the C-terminus. The cut-off score was set to 3.0 for NLS prediction analysis. (TIF) [file pgen.1008620.s021.tif]

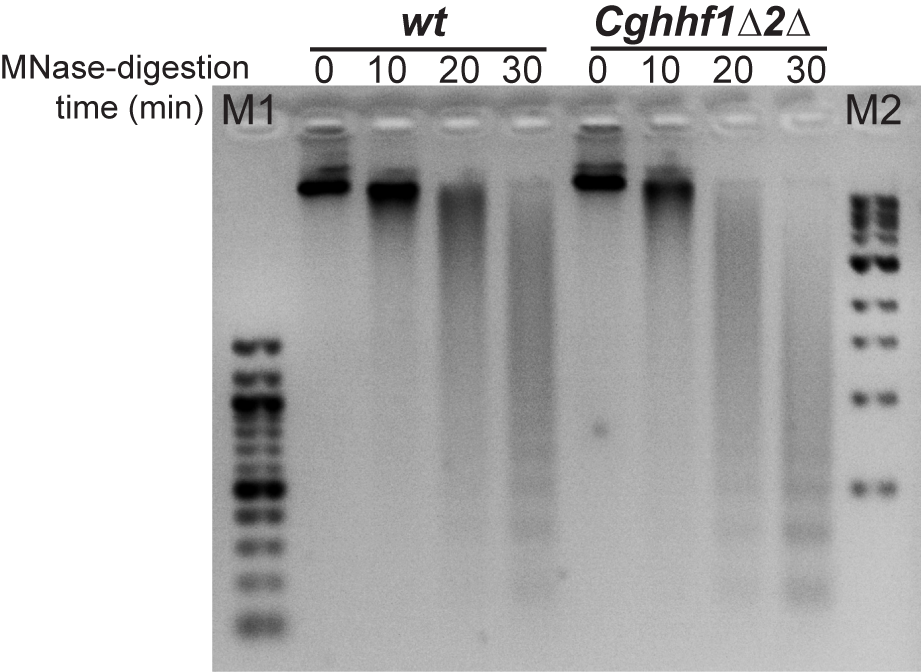

Supplement: S5 Fig — An equal number of log-phase wt and Cghhf1Δ2Δ cells were collected, suspended in spheroplasting buffer [50 mM Tris Cl (pH 7.5) and 1 M sorbitol] and treated with zymolyase (1 mg/10 OD600 cells) for 30 min at 37°C. The generated spheroplasts were suspended in MNase-digestion buffer [10 mM Tris (pH 8.0) and 1 mM CaCl2] and digested with 10 units of MNase at 37°C. Digested samples were harvested at indicated time points, and DNA was isolated using the phenol-chloroform extraction method. 12 μg of purified DNA were resolved on 1.4% agarose gel and stained using ethidium bromide. Marker M1 and M2 indicate 100 bp (NEB #N3231L) and 1 kb (NEB #3232L) DNA ladder, respectively. (TIF) [file pgen.1008620.s022.tif]

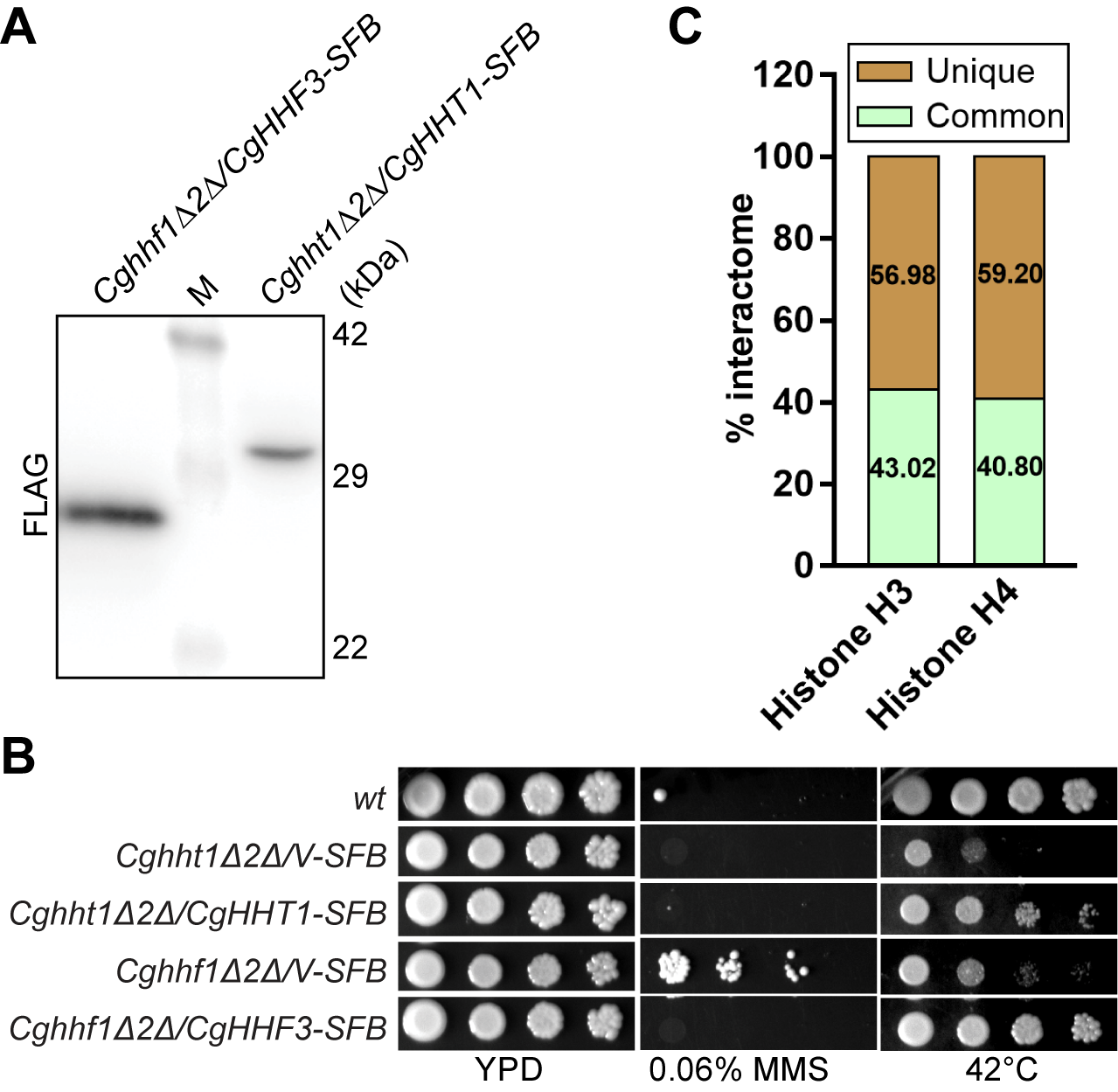

Supplement: S6 Fig — A. An immunoblot showing expression of histone H3 (CgHht1) and H4 (CgHhf3) proteins tagged with the triple SFB epitope at their C-termini. The Cghht1Δ2Δ and Cghhf1Δ2Δ mutants were transformed with plasmids expressing CgHHT1-SFB and CgHHF3-SFB, respectively. Transformants were grown in the CAA medium till log-phase, and whole-cell extracts were prepared by glass bead lysis. 50 μg protein were resolved on 15% SDS-PAGE and probed with anti-FLAG antibody. The bands of 27 kDa and 32 kDa correspond to H4-SFB and H3-SFB histone proteins, respectively. B. Serial dilution spot assay showing that CgHht1-SFB and CgHhf3-SFB could restore the thermal stress sensitivity, and thermal stress sensitivity and MMS resistance of Cghht1Δ2Δ and Cghhf1Δ2Δ mutants, respectively. C. Bar graphs displaying overlap between the interactomes of C. glabrata and S. cerevisiae histone H3 and H4 proteins. The S. cerevisiae interactome information was obtained from the BioGRID interaction database (https://thebiogrid.org/). (TIF) [file pgen.1008620.s023.tif]

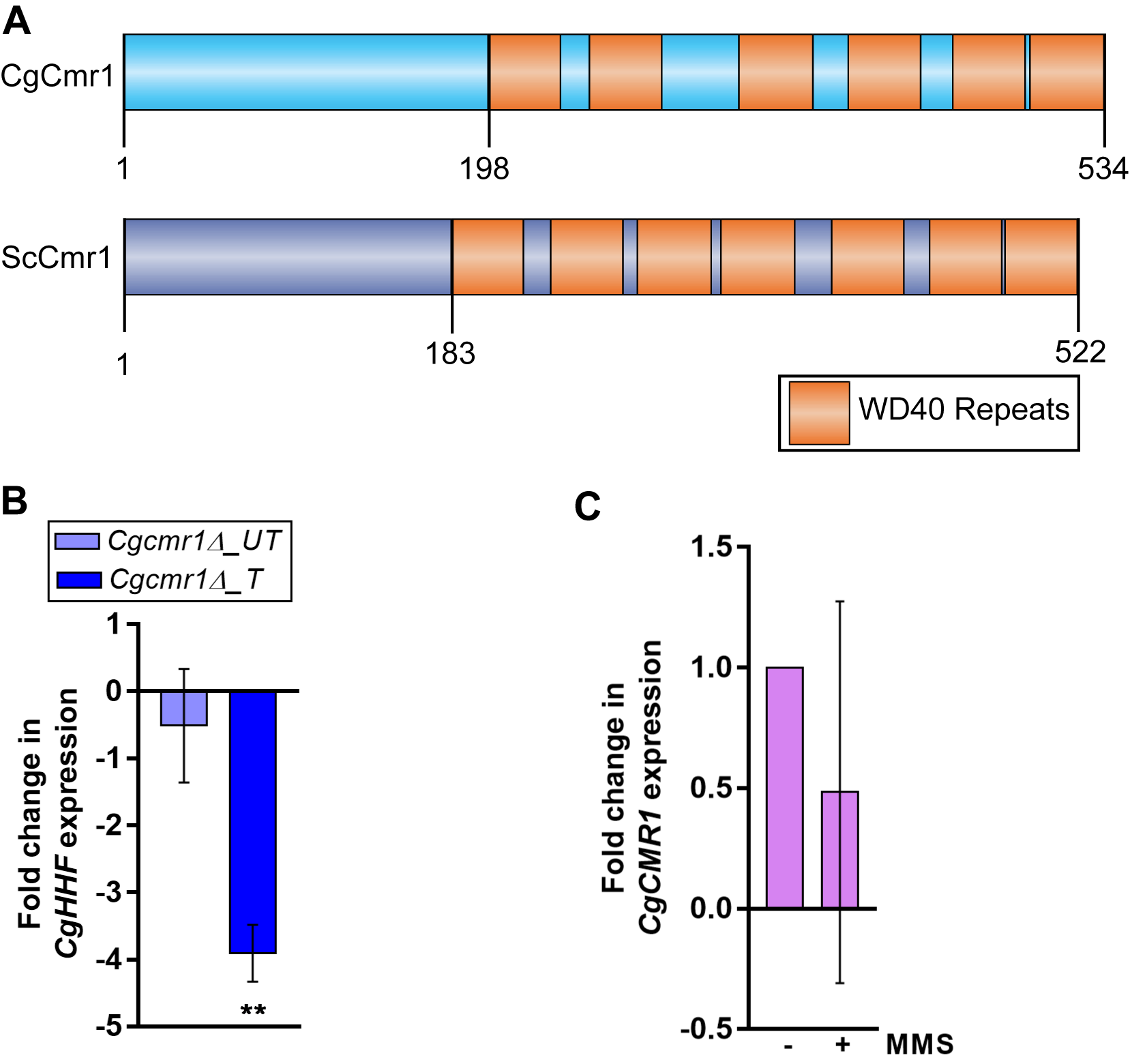

Supplement: S7 Fig — A. Schematic illustration of the domain organization of C. glabrata and S. cerevisiae Cmr1 protein. Information for this analysis was obtained from the Uniprot Database (https://www.uniprot.org). ScCmr1 has seven WD40 repeats, while CgCmr1 protein contains six WD40 repeats. B. qPCR-based measurement of histone H4 transcripts in the untreated and MMS-treated Cgcmr1Δ mutant. YPD medium-grown, log-phase cultures of wt and Cgcmr1Δ strains were either left untreated or treated with 0.06% MMS for 45 min, and RNA was extracted using the acid phenol method. Transcript levels of the CgHHF gene were measured by qPCR. Data (mean ± SEM, n = 3) were normalized against the CgACT1 mRNA control, and represent fold change in H4 gene expression in the Cgcmr1Δ mutant (Cgcmr1Δ_UT) compared to wt cells (considered as 1.0), and in MMS-treated Cgcmr1Δ cells (Cgcmr1Δ_T) compared to untreated Cgcmr1Δ cells (considered as 1.0). **, p ≤ 0.01; paired two-tailed Student’s t test. Please note that CgCMR1 loss had no effect on transcription of the H4 gene. C. qPCR-based measurement of CgCMR1 transcripts in MMS-treated wt cells. Log-phase wt cultures were grown either in the presence or absence of 0.06% MMS for 45 min, and RNA was extracted using the acid phenol method. CgCMR1 transcript levels were measured by qPCR. Data (mean ± SEM, n = 3) were normalized against the CgACT1 mRNA control, and represent fold change in CgCMR1 expression in MMS-treated compared to untreated wt sample. Please note that MMS exposure had no effect on transcription of the CgCMR1 gene. (TIF) [file pgen.1008620.s024.tif]

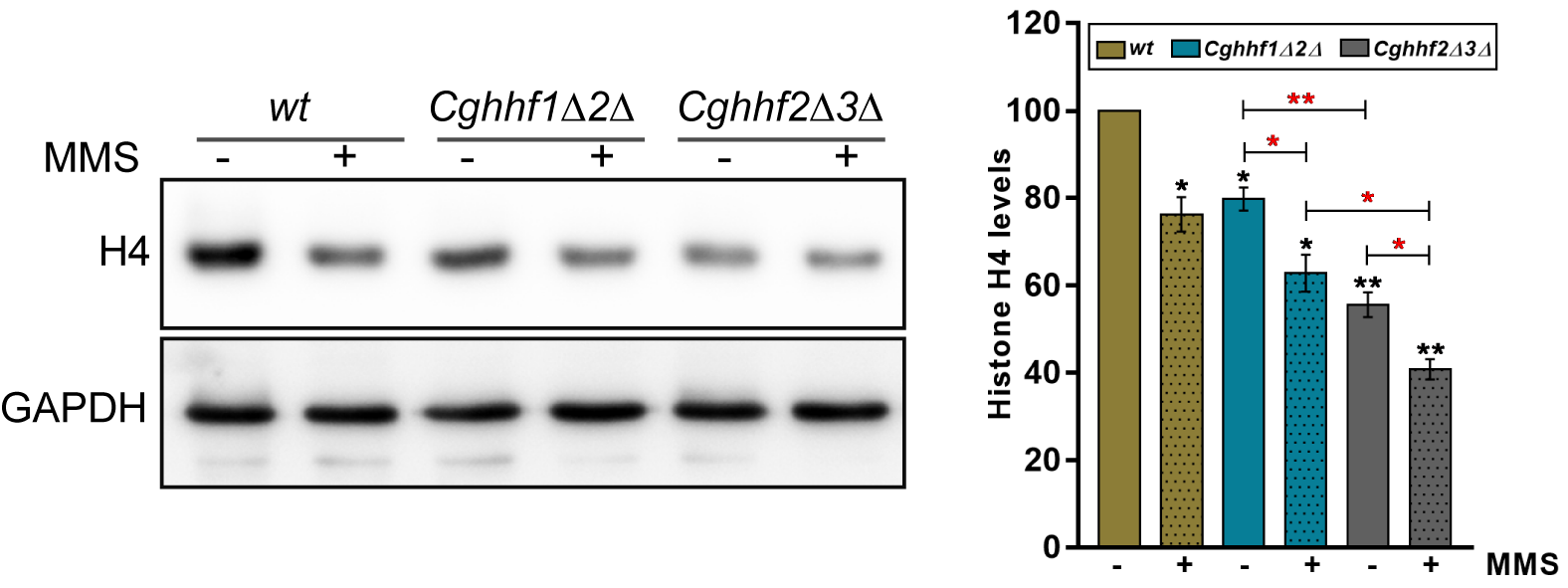

Supplement: S8 Fig — Representative immunoblot showing histone H4 levels in MMS-treated wt, Cghhf1Δ2Δ and Cghhf2Δ3Δ cells. Log-phase cultures were either left untreated or treated with 0.06% MMS for 45 min, whole cell lysates (50 μg) resolved on 15% SDS-PAGE and were probed with anti-H4 and anti-GAPDH antibodies. Data (mean ± SEM, n = 3) represent % change in H4 levels compared to wt untreated samples (considered as 100), and are plotted as a bar graph on the right side of the blot image. *, p ≤ 0.05, **, p ≤ 0.01; paired two-tailed Student’s t test. *, p ≤ 0.05, **, p ≤ 0.01; unpaired two-tailed Student’s t test (Red asterisks). (TIF) [file pgen.1008620.s025.tif]

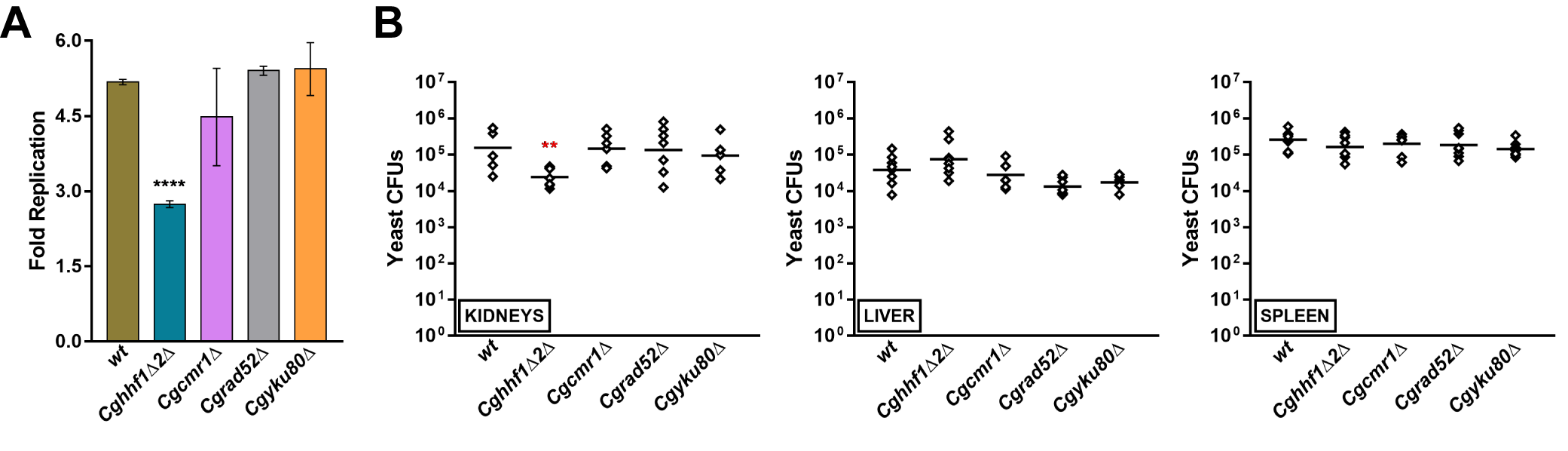

Supplement: S9 Fig — A. Intracellular proliferation of indicated C. glabrata strains in human THP-1 macrophages, as measured by the CFU-based assay. The human monocytic THP-1 cells were treated with phorbol 12-myristate 13-acetate (PMA; 16 nM) for 12 h followed by recovery in the fresh RPMI medium for 12–14 h. YPD-grown overnight cultures of C. glabrata strains were infected to PMA-differentiated THP-1 macrophages at a MoI (multiplicity) of 1:10. After 2 h incubation, the non-internalized C. glabrata cells were washed off with PBS, and infection was continued for another 22 h. Infected macrophages were lysed in water at 2 and 24 h post infection, and appropriate dilutions of macrophage lysates were plated on the YPD medium. The number of colonies, that appeared after 1–2 day incubation at 30°C, was counted. Fold replication for each strain was calculated by dividing the number of intracellular C. glabrata cells recovered at 24 h by that recovered at 2 h. Data represent mean ± SEM (n = 3). ****, p<0.001; unpaired two-tailed Student's t-test. B. Survival analysis of indicated C. glabrata strains in the murine model of systemic candidiasis. C. glabrata cells were grown overnight in the YPD medium, collected, washed and were suspended in PBS. 100 μl cell suspension (4X107 cells) was injected into the tail vein of six to eight-week-old female BALB/c mice. Mice were sacrificed 7th day post infection and kidneys, liver and spleen were collected. After organ homogenization in PBS, appropriate homogenate dilutions were plated on the YPD medium containing penicillin and streptomycin, and fungal load in mouse organs was determined. CFUs recovered from organs of the individual mouse are represented by diamonds, while bars indicate the CFU geometric mean (n = 6–8) for each organ. **, p<0.01; Mann-Whitney test. (TIF) [file pgen.1008620.s026.tif]
